# Synthesis of Curcuminoids and Evaluation of Their Cytotoxic and Antioxidant Properties

**DOI:** 10.3390/molecules22040633

**Published:** 2017-04-14

**Authors:** María Concepción Lozada-García, Raúl G. Enríquez, Teresa O. Ramírez-Apán, Antonio Nieto-Camacho, Juan Francisco Palacios-Espinosa, Zeltzin Custodio-Galván, Olivia Soria-Arteche, Jaime Pérez-Villanueva

**Affiliations:** 1 Departamento de Sistemas Biológicos, División de Ciencias Biológicas y de la Salud, Universidad Autónoma Metropolitana-Xochimilco (UAM-X.), Ciudad de México 04960, Mexico; mclozada@correo.xoc.uam.mx (M.C.L.-G.); jpalacios@correo.xoc.uam.mx (J.F.P.-E.); zeltzin_888@hotmail.com (Z.C.-G.); 2 Instituto de Química, Universidad Nacional Autónoma de México (UNAM), Ciudad de México 04510, Mexico; enriquezhabib@gmail.com (R.G.E.); mtrapan@yahoo.com.mx (T.O.R.-A.); anieto@unam.mx (A.N.-C.)

**Keywords:** antioxidant, azoles, cytotoxic, curcumin

## Abstract

Curcumin (**1**) and ten derivatives (**2**–**11**) were synthesized and evaluated as cytotoxic and antioxidant agents. The results of primary screening by Sulforhodamine B assay against five human cancer cell lines (U-251 MG, glioblastoma; PC-3, human prostatic; HCT-15, human colorectal; K562, human chronic myelogenous leukemia; and SKLU-1, non-small cell lung cancer) allowed us to calculate the half maximal inhibitory concentration (IC_50_) values for the more active compounds against HCT-15 and K562 cell lines. Compounds **2** and **10** were the most active against both cell lines and were more active than curcumin itself. Thiobarbituric acid reactive substances (TBARS) assay showed that **7** has potent activity; even stronger than curcumin, α-tocopherol, and quercetin.

## 1. Introduction

Curcumin, a natural yellow pigment present in the rhizomes of several curcuma species, has been employed in different Asian countries as a dietary condiment, and it has been used for the treatment of a variety of diseases in traditional Chinese and Indian medicine [1]. Recent studies have been published on the broad biological activities of curcumin [1,2], which include its anti-inflammatory, anti-oxidant, antimicrobial, antiviral, anti-cystic fibrosis, and anticancer properties [2,3]. Preclinical studies have suggested curcumin as a drug candidate, and considerable research efforts have been carried out to understand its mechanisms of action [4]. Furthermore, curcumin has been considered an epigenetic agent, which acts by modulating multiple biological processes at low concentration [5]. This molecule can bind as many as 33 different proteins, including targets such as transcriptional factors, growth factors, cytokines, and genes regulating cell proliferation and apoptosis [1,5]. However, the clinical potential of curcumin is limited by its extremely low oral bioavailability, due to its poor aqueous solubility and its chemical instability [6]. Several strategies have been proposed to overcome the low bioavailability of curcumin and increase its solubility. These include the use of adjuvants that block metabolic pathways, encapsulation with nanoparticles [7], liposomes [8], cyclodextrins [9], bile salt aggregates as carrier [10], etc. An additional strategy has been the synthesis of analogues or derivatives to increase its bioavailability or improve certain biological activities. In spite of its drawbacks, curcumin has been considered a promising lead structure [11] to design new derivatives with enhanced properties, due to its safety [1,3] and pharmacological potential.

In the last decades, a broad number of analogues and derivatives of curcumin have been synthesized. These new compounds have shown improved effects as free radical scavengers [12], cytotoxic agents [13,14], anti-inflammatories, and others [15]. Some also have antitumoral properties, behaving as antitumor agents in multidrug resistant cancer cells [16], NF-kB activators [17], antioxidants [18], pro-oxidants [19,20], and antivirals [21].

Curcumin is a diarylheptanoid, with two *o*-methoxyphenols attached to a β-diketone by two symmetrical olefinic bonds. Several modifications have been made to the curcumin structure, including derivatives with 1,2-azole rings instead of the β-diketone [6]. It is worth mentioning that azole curcumin derivatives have shown to be stable at physiological pH, having good antiproliferative activity against several cancer cell lines and better free radical scavenging activity than its predecessor [6,11,14,16,22,23]. Several reports have suggested that the anti-oxidant activity of curcumin and its derivatives depends upon the presence of phenolic groups [24,25].

**Figure 1:**
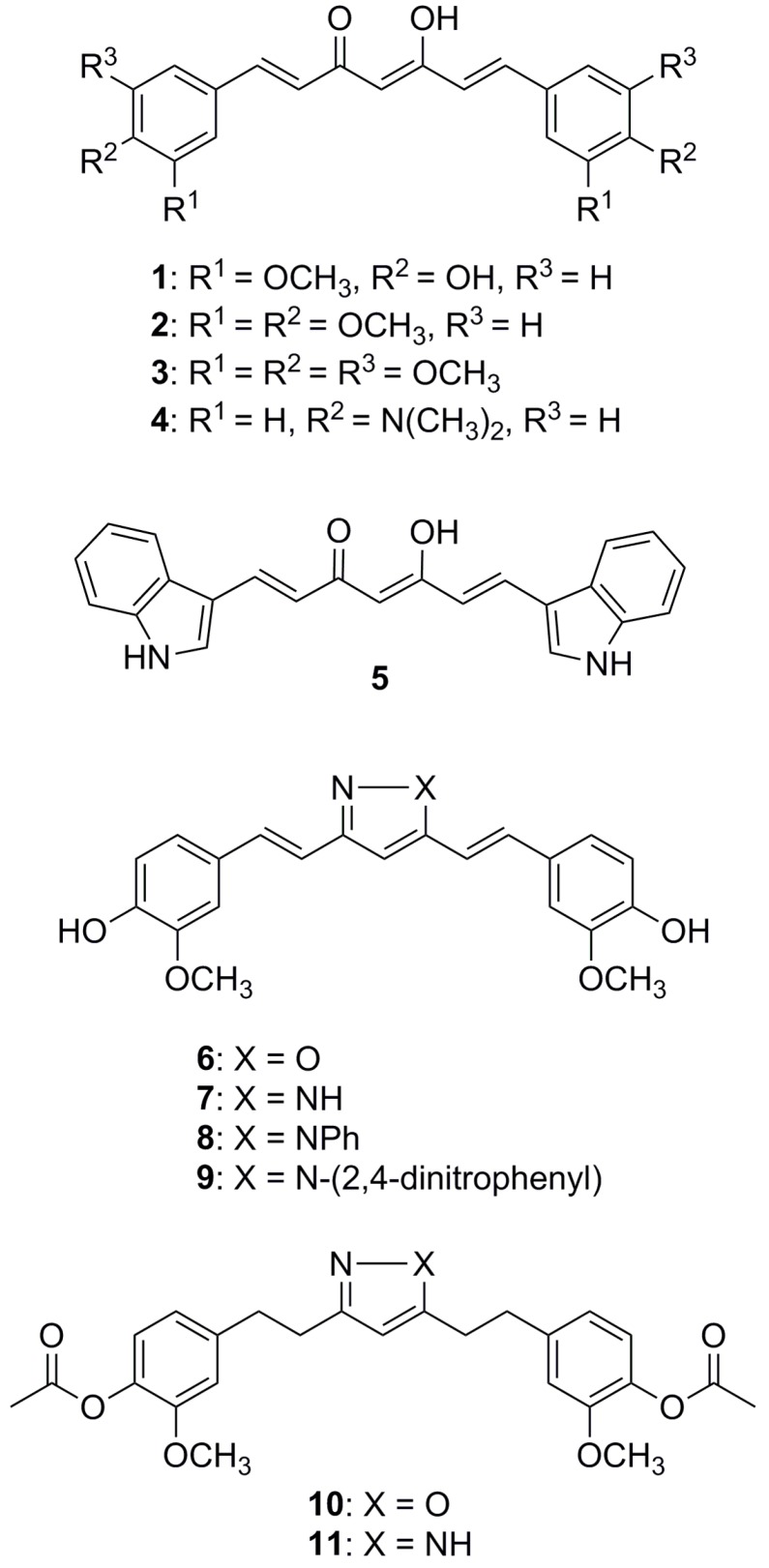
Chemical structure of curcumin and the analogues synthesized.

Herein we synthesized curcumin **1** and ten of its derivatives, **2**–**11**, to expand the knowledge about the biological activity of curcuminoids as cytotoxic and antioxidants agents (Figure 1). All compounds were evaluated as cytotoxic agents in five human cancer cell lines. In view of the previous assays, the most active compounds were also tested as antioxidants using the 2,2-diphenyl-1-picrylhydrazyl (DPPH) and thiobarbituric acid reactive substances (TBARS) models.

## 2. Results and Discussion

### 2.1. Synthesis of Curcumin Derivatives

**Scheme 1:**
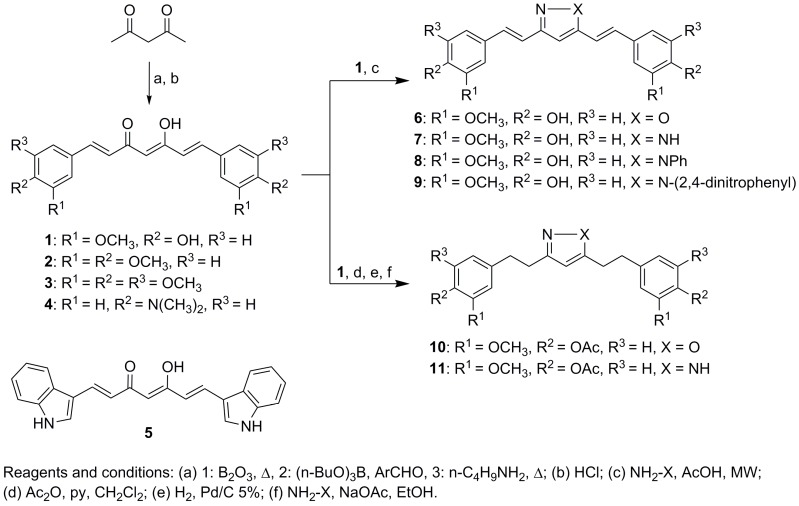
Synthesis of curcumin derivatives **1**–**11**.

Compounds **1**–**5** were synthesized by aldol condensation of the corresponding substituted aromatic aldehyde with 2,4-pentanodione and butylamine, by a slight modification of Pabon synthesis (Scheme 1) [11,26]. First, a complex with boric anhydride and 2,4-pentanodione was formed; then, a mixture of arylaldehyde and tributylborate was added, followed by addition of *n*-butylamine under N_2_ atmosphere. Finally, the resulting boron complex was treated in situ with acetic acid to obtain the respective curcumin derivative in moderate yields. The synthesis of azole derivatives of curcumin is shown in Scheme 1. The isoxazole derivative of curcumin **6** was prepared by condensation of curcumin with hydroxylamine hydrochloride in acetic acid at 120 °C, using a microwave monomodal reactor under flash heating conditions in sealed vessels [27]. Pyrazole derivatives of curcumin **7**–**9** were prepared in a similar way by heating curcumin with hydrazine hydrate, phenyl hydrazine, and 2,4-dinitrophenylhydrazine, respectively. As reported by previous work [28], the synthesis of azoles of curcumin heated under microwave irradiation decreased the reaction time and in some cases increased reaction yields.

The azole derivatives **10** and **11** were synthesized as described previously [29], by acetylation of curcumin with acetic anhydride and pyridine, followed by a catalytic hydrogenation with Pd/C (5%) and cyclization with hydroxylamine hydrochloride or hydrazine sulfate in EtOH with NaOAc, respectively. The structure of all synthesized compounds was confirmed using spectroscopic and spectrometric methods. Additionally, melting points were determined and they were consistent with the literature reports. The nuclear magnetic resonance and mass spectra of compounds can be found in Supplementary Materials.

### 2.2. Biological Assays

#### 2.2.1. Cytotoxic Activity against Human Cancer Cell Lines

It is known that curcumin and several curcuminoids possess anti-proliferative activity [14,23,30]. Therefore, these derivatives also have been considered as potential anti-tumor agents [14]. However, the knowledge of biological properties of curcumin and its derivatives can be expanded through the exploration of their cytotoxic activity in other cell lines.

The cytotoxic activity of the synthesized compounds was evaluated employing the Sulforhodamine B assay against five human cancer cell lines: U-251 MG (glioblastoma), PC-3 (human prostatic), HCT-15 (human colorectal), K562 (human chronic myelogenous leukemia), and SKLU-1 (non-small cell lung cancer). Curcumin (**1**) and its derivatives **2**–**11** were screened at 50 µM concentration [31]. The results of growth inhibition for the five cell lines tested are shown in Table 1. Since compounds **1**–**2**, and **5**–**7** exhibit high activity at 50 µM (100% of growth inhibition), they were subsequently tested at lower concentrations of 12.5 and 10 µM, as indicated in Table 1. Compounds **1**, **2**, **5**–**8**, and **10**–**11** showed strong activity against most of the cancer cell lines tested (% growth inhibition > 50), but particularly on HCT-15 and K562.

Results from the primary screening allowed us to calculate the inhibitory concentration 50 (IC_50_) values for the more active compounds against K562 and HCT-15 cell lines. Although several studies have reported cytotoxic activity for curcumin derivatives, including some of the compounds reported here [14,23,30], no reports were available for compounds **1**–**11** against K562 and HCT-15 cell lines. The calculated IC_50_ values are shown in Table 2. The results for the HCT-15 cancer cell line show that all selected compounds (**2**, **5**, **6**, **7**, **8**, **10**, and **11**) were more active than curcumin (**1**). The most active compound was **2** with IC_50_ = 2.3 μM, which is 6-fold more active than curcumin. According to these results, the methylation of the hydroxyl group at the aromatic ring in compound **2** improved the cytotoxic effects, however, this was the only successful modification on the aromatic ring that improved the activity. The introduction of an additional methoxy (compound **3**) and the presence of an *N,N*-dimethylamine group (compound **4**) decreased the cytotoxic activity in both cases (Table 1). On the other hand, the substitution of aromatic rings by 3-indolyl moieties (compound **5**) increased the cytotoxic effect relative to that of curcumin. To explore the effect of the replacement of β-diketone for a 1,2-azole ring, compounds **6**, **7**, and **8** were tested. The results show that substitution of diketone for pyrazole, *N*-phenylpyrazole, and oxazole improves the cytotoxic activity compared with that of curcumin, but no differences in potency were found among them. Therefore, 1,3-diketone is not essential for the cytotoxic activity, at least in this compound series. A similar result has been reported for some curcuminoids tested against other different cancer cell lines [6,23]. Last, compounds **10** and **11** are more active than curcumin, however, the oxazole derivative **10** is more active than the pyrazole analog **11** against HCT-15 cell line (Table 2). It is worth noting that **10** and **11** lack the conjugated double bounds and the 1,3-diketone, therefore, these characteristics are not essential to obtain active compounds. For assays on K562 cancer cell line, the activity changes in compounds **1**, **2**, **7**, **8**, **10**, and **11** show the same tendency as that observed against the HCT-15 cell line. Compound **2** showed the most important cytotoxic effect against K562, with IC_50_ = 2.3 μM, which is 4-fold more active than curcumin. Also, compounds **7**, **8**, and **10** were more active than curcumin. However, some differences were found when compared with the results from the HCT-15 cell line. At this point, inverse structure–activity relationships (SAR) were found for compounds **5** and **6**; whereas structural modifications increase the activity against HCT-15 relative to curcumin, the same modifications decrease the activity against K562. Although the compounds tested display cytotoxic activity in the low micromolar range against K562 and HCT-15, in most cases higher than that of curcumin, they have lower potency than the Adriamycin standard.

#### 2.2.2. Antioxidant Activity in the Thiobarbituric Acid Reactive Substances and 2,2-Diphenyl-1-picrylhydrazyl Assays

Based on the results of the previous assays, curcumin and the most active compounds as cytotoxic agents (**2** and **10**) were selected to test for antioxidant activity. Compounds **6** and **7** were also selected since no reports on the TBARS assay were available, and they exhibited significant cytotoxic activity, at least on one of the cell line tested. Two different in vitro assays were used to evaluate the antioxidant activity of compounds [31]. The TBARS assay measures the amount of malondialdehyde (MDA) as a product of lipid oxidation and the DPPH measures the efficacy of a hydrogen atom transferred from the compound to the radical by colorimetric determination. Quercetin and α-tocopherol were used as reference antioxidants. As shown in Table 3, curcumin possess IC_50_ values of 3.22 and 23.52 µM in TBARS and DPPH assays, respectively; which agrees with previous literature reports [32,33]. Compounds **2** and **10** showed only weak activity in the TBARS assays, which in turn suggests that these compounds exhibit their antioxidant activity by a non-radical scavenging mechanism. On the other hand, compounds **6** and **7** had strong activity in the TBARS assay. Although the antioxidant activity using the DPPH assay for these compounds was previously reported by Sherin and Rajasekharan, the same assay was performed in this work to compare experimentally both methods (DPPH and TBARS) [34]. The values found in the DPPH assay compared to the results in the TBARS assay show that compounds **6** and **7** exhibit an antioxidant activity mainly by lipid peroxidation inhibition. A similar conclusion can be achieved if values for DPPH assay reported by Sherin and Rajasekharan are taken as a reference [34].

## 3. Experiment Sections

### 3.1. Chemistry

#### 3.1.1. General Considerations

Microwave assisted reactions were carried out in a monomodal reactor equipped with a hydraulic pressure sensing device and an immersing ruby thermometer for precise control of reaction temperature (Anton Parr Monowave 300, Anton Parr, Graz, Austria). Melting points (°C) were determined in capillary Pyrex using Buchi Melting Point M-565 (Buchi, Flawil, Switzerland). Mass spectra were obtained in a JEOL JMS-AX505HA mass spectrometer (Jeol, Tokyo, Japan) by electronic impact at 70 eV and JEOL SX 102, whereas spectra using electrospray ionization (ESI) were recorded on a Bruker ESI/APCI-TOF (MicroTOF-II-Focus) spectrometer (Bruker, Billerica, MA, USA). ^1^H- and ^13^C-NMR spectra were recorded either on 200, 300, 400, or 600 MHz Varian NMR spectrometer (Palo Alto, CA, USA) or 300 or 500 MHz Bruker NMR spectrometer (Bruker, Billerica, MA, USA), using tetramethylsilane (TMS) as the internal standard. Chemical shifts (δ) are given in ppm and coupling constants (*J*) in Hz. TLC (Thin Layer Chromatography) was performed on precoated Kieselgel 60 F_254_ plates (Merck, Darmstadt, Germany) and detected with ultraviolet (UV) light. Flash chromatography was performed with Merck Kieselgel silica gel (60, particle size 40–63 mm) (Merck, Kenilworth, NJ, USA). All reagents were purified and the solvents used were of analytical grade. NMR spectra of compounds **1**–**11** and mass spectra of compounds **1**–**2** and **4**–**11** can be found in Supplementary Materials.

#### 3.1.2. General Procedure for the Preparation of **1**, **2**, **4**, and **5**

2,4-Pentanodione (0.204 g, 2.04 mmol) and boric anhydride (0.071 g, 1.02 mmol) were dissolved in anhydrous ethyl acetate (EtOAc) (4 mL). The solution was stirred for 3 h at 70 °C under N_2_ atmosphere. Then, the mixture was added to a solution of arylaldehyde (4.9 mmol) and tributyl borate (1.12 g, 4.9 mmol) in anhydrous EtOAc (10 mL) which was stirred for one hour under N_2_ atmosphere; then, *n*-butylamine (0.245 mmol) dissolved in anhydrous EtOAc (5 mL) was added dropwise. The reaction mixture was stirred for 10–24 h at 70 °C and was quenched by addition of 1 N HCl (20 mL). The reaction mixture was stirred at 70 °C for one hour and the aqueous layer was extracted with EtOAc (4 × 20 mL). The combined organic layers were washed with brine. The organic phase was dried with Na_2_SO_4_, filtered, and the solvent was removed in vacuo. The residue was purified by column chromatography (SiO_2_, Hexane/EtOAc, 30:70) to give **1**, **2**, **4**, and **5**, respectively.

*(1E,4Z,6E)-5-Hydroxy-1,7-bis(4-hydroxy-3-methoxyphenyl)hepta-1,4,6-trien-3-one* (**1**). Red light solid (69% yield); m.p.: 180–183 °C (lit. [24] 182–184 °C); ^1^H-NMR (500 MHz, CDCl_3_) δ: 16.105 (bs, 1H), 7.745 (bs, 2H), 7.574 (d, 2H, *J* = 15.5 Hz), 7.079 (dd, 2H, *J* = 8.0, 2 Hz), 7.061 (d, 2H, *J* = 1.5 Hz), 6.916 (d, 2H, *J* = 8 Hz), 6.473 (d, 2H, *J* = 15.5 Hz), 5.814 (s, 1H), 3.933 (s, 6H); ^13^C-NMR (125 MHz, CDCl_3_) δ: 183.053, 148.494, 147.32, 145.338, 140.438, 126.955, 122.68, 121.134, 115.205, 109.916, 100.878, 55.731; EIM *m*/*z* (rel. int.): 368 (M^+^, 62%).

*(1E,4Z,6E)-5-Hydroxy-1,7-bis(3,4-dimethoxyphenyl)hepta-1,4,6-trien-3-one* (**2**). Dark yellow solid (55% yield); m.p.: 129–130 °C (lit. [24] 129–131 °C); ^1^H-NMR (200 MHz, CDCl_3_) δ: 7.609 (d, 2H, *J* = 15.8 Hz), 7.147 (dd, 2H, *J* = 8.2, 1.6 Hz), 7.078 (d, 2H, *J* = 2.0 Hz), 6.881 (d, 2H, *J* = 8.4 Hz), 6.498 (d, 2H, *J* = 15.8 Hz); 5.824 (s, 1H), 3.938 (s, 6H), 3.926 (s, 6H); ^13^C-NMR (50 MHz, CDCl_3_) δ: 183.195, 150.968, 149.147, 140.377, 127.981, 122.625, 121.957, 111.033, 109.622, 101.277, 55.955, 55.864; EIM (*m*/*z*) 396 (38%).

*(1E,4Z,6E)-5-Hydroxy-1,7-bis(3,4,5-trimethoxyphenyl)hepta-1,4,6-trien-3-one* (**3**). 2,4-Pentanodione (0.127 g, 1.27 mmol), boric anhydride (0.044 g, 0.63 mmol), trimethoxybenzaldehyde (0.5 g, 2.55 mmol), tributyl borate (0.586, 2.55 mmol, 0.68 mL), and *n*-butylamine (0.0186 g, 0.25 mmol, 0.13 mL) were dissolved in *N*,*N*-dimethylformamide (3 mL) in a 10 mL reaction vial. The reaction mixture was heated to 100 °C under microwave irradiation for 2 min at 400 rpm. After completion of the reaction, the mixture was poured into ice-water (50 mL) and filtered to give **3** as an orange solid (40% yield); m.p.: 184–185 °C (lit. [35] 189–190 °C); δ: ^1^H-NMR (500 MHz, CDCl_3_) δ: 15.907 (bs, 1H), 7.579 (d, 2H, *J* = 16.0 Hz), 6.782 (s, 4H), 6.527 (d, 2H, *J* = 15.5 Hz), 5.863 (s, 1H), 3.907 (s, 12H), 3.893 (s, 6H); ^13^C-NMR (125 MHz, CDCl_3_) δ: 183.085, 153.442, 145.432, 140.572, 140.102, 130.485, 123.362, 105.299, 101.508, 60.977, 56.158.

*(1E,4Z,6E)-1,7-bis(4-(Dimethylamino)phenyl)-5-hydroxyhepta-1,4,6-trien-3-one* (**4**). Red solid (50% yield); m.p.: 213–215 °C (lit. [36] 212–214 °C); ^1^H-NMR (500 MHz, CDCl_3_) δ: 16.344 (bs, 1H), 7.587 (d, 2H, *J* = 16.0 Hz), 7.438 (d, 4H, *J* = 8.5 Hz), 6.665 (d, 4H, *J* = 9.0 Hz), 6.407 (d, 2H, *J* = 16.0 Hz), 5.714 (s, 1H), 3.009 (s, 12H); ^13^C-NMR (125 MHz, CDCl_3_) δ: 183.277, 151. 552, 145.766, 140.534, 130.517, 129.774, 123.032, 120.671, 119.129, 111.893, 100.831, 40.119; EIM *m*/*z* (rel. int.): 362 (M^+^, 84%).

*(1E,4Z,6E)-5-Hydroxy-1,7-di(1H-indol-3-yl)hepta-1,4,6-trien-3-one* (**5**). Red solid (58% yield); m.p.: 204–207 °C (lit. [37] 210 °C); ^1^H-NMR (600 MHz, DMSO-*d_6_*) δ: 10.731 (s, 2H), 6.914 (d, 2H, *J* = 7.8 Hz), 6.863 (d, 2H, *J* = 2.4 Hz), 6.794 (d, 2H, *J* = 15.6 Hz), 6.417 (d, 2H, *J* = 7.8 Hz), 6.179–6.127 (m, 4H), 6.156 (d, 2H, *J* = 15.6 Hz), 5.076 (s, 1H); ^13^C-NMR (150 MHz, DMSO-*d_6_*) δ: 183.448, 137.682, 134.603, 132.033, 125.143, 122.877, 121.213, 120.276, 118.431, 112.952, 112.665, 100.262; EIM *m*/*z*: 354 (M^+^ 8%).

#### 3.1.3. Procedure for the Synthesis of Isoxazole Derivative **6**

*4,4′-((1E,1'E)-Isoxazole-3,5-diylbis(ethene-2,1-diyl))bis(2-methoxyphenol*) (**6**). To a solution of curcumin **1** (0.258 g, 0.7 mmol) in 5 mL of glacial acetic acid, hydroxylamine hydrochloride (0.073 g, 1.05 mmol) was added. The reaction mixture was heated to 70 °C with microwave irradiation for 5 min stirring at 400 rpm. Then, the reaction mixture was dropped into water to precipitate, filtered in vacuo, and washed with cold water. The solid was purified by recrystallization (MeOH) to give compound **6** (98% yield); m.p. 163–164 °C (lit. [28] 162 °C); ^1^H-NMR (600 MHz, CDCl_3_) δ: 16.034 (bs, 2H), 7.591 (d, 2H, *J* = 15.6 Hz), 7.124 (dd, 2H, *J* = 1.8, 7.8 Hz), 7.052 (d, 2H, *J* = 1.2 Hz), 6.936 (d, 2H, *J* = 8.4 Hz), 6.478 (d, 2H, *J* = 15.6 Hz), 5.857 (s, 1H), 3.951 (s, 6H); ^13^C-NMR (125 MHz, CDCl_3_) δ: 183.479, 148.061, 146.999, 140.748, 127.921, 123.092, 122.008, 115.047, 109.852, 101.386, 56.194. EIM *m*/*z* (rel. int.): 365 (M^+^, 100%).

#### 3.1.4. General Procedure for the Preparation of Pyrazole Analogues of Curcumin (**7**–**9**)

To a solution of curcumin **1** (0.105 g, 0.27 mmol) in glacial acid acetic (3 mL), hydrazine hydrate (0.405 mmol), phenylhydrazine (0.324 mmol), or 2,4-dinitrophenylhydrazine (0.27 mmol) were added. The reaction mixture was heated to 120 °C under microwave irradiation for 10 min and stirred at 400 rpm. The reaction mixture was dropped into water to precipitate, then filtered in vacuo, and washed with cold water. The solid was purified by recrystallization.

*4,4′-((1E,1′E)-(1H-Pyrazole-3,5-diyl)bis(ethene-2,1-diyl))bis(2-methoxyphenol)* (**7**). Light brown solid (EtOH/H_2_O) (71% yield); m.p.: 218–220 °C (lit. [38] 218 °C); ^1^H-NMR (500 MHz, CDCl_3_) δ: 8.067 (bs, 2H), 7.573 (d, 2H, *J* = 15.5 Hz), 7.387 (s, 1H), 7.075 (d, 2H, *J* = 7.0 Hz), 7.068 (s, 2H), 6.914 (d, 2H, *J* = 8.5 Hz), 6.477 (d, 2H, *J* = 15.5 Hz), 5.82 (s, 1H), 3.936 (s, 6H); ^13^C-NMR (125 MHz, CDCl_3_) δ: 182.979, 148.580, 147.374, 140.385, 122.620, 120.987, 115.867, 115.324, 109.908, 100.797, 55.646. 183.197, 148.798, 147.592, 140.603, 126.99, 122.838, 121.206, 115.452, 110.126, 101.015, 55.864; EIM *m*/*z* (rel. int.): 364 (M^+^, 94%).

*4,4′-((1E,1′E)-(1-Phenyl-1H-pyrazole-3,5-diyl)bis(ethene-2,1-diyl))bis(2-methoxyphenol)* (**8**). Light yellow solid (EtOH/H_2_O) (94% yield); m.p.: 104–105 °C (lit. [28] 89 °C), (lit. [39] 128–129 °C); ^1^H-NMR (600 MHz, CDCl_3_) δ: 7.538 (d, 2H, *J* = 8.4 Hz), 7.504 (t, 2H, *J* = 7.8 Hz), 7.412 (t, 1H, *J* = 7.2 Hz), 7.125 (d, 1H, *J* = 16.8 Hz), 7.086 (d, 1H, *J* = 1.2 Hz), 7.054 (d, 1H, *J* = 16.2 Hz), 7.038 (d, 1H, *J* = 16.2 Hz), 7.002 (dd, 1H, *J* = 8.4, 1.8 Hz), 6.964 (dd, 1H, *J* = 8.4, 1.8 Hz), 6.897 (t, 3H, *J* = 8.4 Hz), 6.814 (s, 1H), 6.717 (d, 1H, *J* = 16.2 Hz), 3.923 (s, 3H), 3.893 (s, 3H); ^13^C-NMR (150 MHz, CDCl_3_) d: 151.589, 146.971, 146.462, 145.949, 142.757, 139.798, 132.566, 130.761, 129.996, 129.415, 129.217, 128.013, 125.417, 120.099, 120.785, 118.516, 114.934, 114.719, 113.662, 108.915, 108.081, 100.638, 56.177, 56.061; EIM *m*/*z* (rel. int.): 440 (M^+^, 100%).

*4,4′-((1E,1′E)-(1-(2,4-Dinitrophenyl)-1H-pyrazole-3,5-diyl)bis(ethene-2,1-diyl))bis(2-methoxyphenol)* (**9**). Dark orange solid (MeOH/CH_2_Cl_2_) (60% yield); m.p.: 212–213 °C (lit. [39] 203–204 °C); ^1^H-NMR (600 MHz, CDCl_3_) δ: 8.827 (d, 1H, *J* = 2.4 Hz), 8.57 (dd, 1H, *J* = 8.4, 2.4 Hz), 7.832 (d, 1H, *J* = 9.0 Hz), 7.449 (bs, 1H), 7.169 (bs, 1H), 7.114 (d, 1H, *J* = 16.2 Hz), 7.097 (d, 1H, *J* = 15.6 Hz), 7.065 (d, 1H, *J* = 1.8 Hz), 6.979 (dd, 1H, *J* = 8.4, 1.8 Hz), 6.932 (dd, 1H, *J* = 8.4, 1.8 Hz), 6.899 (d, 1H, *J* = 15.6 Hz), 6.90–6.87 (m, 4H), 6.522 (d, 1H, *J* = 15.6 Hz), 3.934 (s, 3H), 3.903 (s, 3H); ^13^C-NMR (150 MHz, CDCl_3_ + DMSO-*d*_6_) δ: 154.241, 147.573, 147.48, 147.385, 146.742, 146.148, 145.303, 143.981, 137.46, 135.344, 132.75, 129.527, 128.823, 127.822, 127.542, 121.253, 120.990, 120.949, 116.773, 115.395, 115.098, 110.337, 109.517, 108.601, 102.244, 56.068, 55.945; MS (HR–ESI) for C_27_H_22_N_4_O_8_ [M + H]^+^, calcd.: *m*/*z* 531.1510, found: *m*/*z* 531.1512.

#### 3.1.5. Synthesis of Compounds **10** and **11**

The synthesis of compounds **10** and **11** were carried employing the methodology previously described by Lozada et al. [29].

*(Isoxazole-3,5-diylbis(ethane-2,1-diyl))bis(2-methoxy-4,1-phenylene) diacetate* (**10**). White solid (CH_2_Cl_2_/CH_3_OH) (80% yield); m.p. 80–82 °C (lit. [29] 80–82 °C); ^1^H-NMR (300 MHz, CDCl_3_): 2.3 (s, 6H), 2.94 (s, 4H), 2.99 (2t, *J* = 4.5, 4H), 3.79 (s, 3H), 3.80 (s, 3H), 5.73 (s, 1H), 6.73–6.79 (m, 2H), 6.81 (d, *J* = 1.8, 2H), 6.94 (d, *J* = 7.8, 2H); ^13^C-NMR (75 MHz, CDCl_3_): 20.62, 27.83, 34.26, 28.44, 33.47, 55.78, 101.14, 112.46, 112.55, 120.30, 120.39, 122.60, 122.66, 138.07, 138.19, 139.02, 139.64, 150.89, 163.04, 169.10, 169.13, 171.91. MS *m*/*z* (rel. int. %): 453 M^+^ (7), 411 [M − 42]^+^ (100), 381 [M − 72]^+^ (8), 369 [M − 84]^+^ (97), 137 (96), 43 (8).

*(1H-Pyrazole-3,5-diyl)bis(ethane-2,1-diyl))bis(2-methoxy-4,1-phenylene) diacetate* (**11**). White solid (CH_2_Cl_2_/CH_3_OH) (80% yield); m.p. 77–79 °C (lit. [29] 77–79 °C); m.p.; ^1^H-NMR (300 MHz, CDCl_3_): 2.30 (s, 6H), 2.92 (s, 8H), 3.77 (s, 6H), 5.86 (s, 1H), 6.73 (dd, 2H, *J* = 8.1, 2.1), 6.76 (d, *J* = 1.8, 2H), 6.92 (d, *J* = 8.1, 2H); ^13^C-NMR (75 MHz, CDCl_3_): 20.63, 28.65, 35.48, 55.76, 102.74, 112.58, 120.37, 122.53, 140.03, 137.98, 148.07, 150.79, 169.22; MS *m*/*z* (rel. int. %): 452 M^+^ (10), 410 [M − 42]^+^ (100), 368 [M − 84]^+^ (56), 274 [M − 178]^+^ (21), 232 (43), 137 (84), 43 (6).

### 3.2. Biological Assays

#### 3.2.1. Cytotoxic Activity 

The cytotoxicity of **1**–**11** on tumor cells was determined using the protein-binding dye sulforhodamine B (SRB) assay in micro culture to determine cell growth [31]. The National Cancer Institute (NCI), USA, supplied the cell lines employed in these experiments (PC-3, K562, HCT-15, and SKLU-1). The cell lines were cultured in RPMI-1640 culture medium (Roswell Park Memorial Institute medium) with 10% fetal bovine serum (FBS), 2 mM L-glutamine, 10,000 units/mL penicillin G, 10,000 µg/mL streptomycin sulfate, and 25 µg/mL amphotericin B (GIBCO). They were kept at 37 °C in an atmosphere of 5% CO_2_ with 95% humidity. With the exception of K562 cell line, the rest of the adherent cell lines were removed from the tissue culture flask by adding 1 mL of 0.05% trypsin-ethylenediaminetetraacetic acid (GIBCO laboratories) and diluted with fresh media. The viability of the cells used in the experiments exceeded 95% as determined with trypan blue. For the assays, 100 µL/well of 5 × 10^4^ cell/mL (K562), 7.5 × 10^4^ cell/ mL (PC-3, SKLU-1), and 10 × 10^4^ cell/mL (HCT-15) of cell suspension were seeded in 96-well micro-titer plates and incubated to allow for cell attachment. Test samples were dissolved in DMSO to provide stock solutions (20 mM). After 24 h of incubation, 100 µL of each tested compound (**1**–**11**) and the reference substance (Adriamycin) was added to each well. After 48 h, adherent cell culture was fixed in situ by adding 50 µL of cold 50% (*w*/*v*) trichloroacetic acid (TCA) and incubated for 60 min at 4 °C. For K562, the suspension cell lines were fixed to the bottom of a microtiter well by gently adding 50 µL of 80% cold TCA. The supernatant was discarded and the plates were washed three times with water and air-dried. Cultures fixed with TCA were stained for 30 min with 100 µL of 0.4% SRB solution. Protein-bounded dye was extracted with 10 mM of unbuffered tris base (tris(hydroximethyl)aminomethane) and the optical density was measured on an Ultra Microplate Reader (Synergy/HT, BIO-TEK Instrument, Inc., Winooski, VT, USA) with a tested wavelength of 515 nm. Results were expressed as half maximal inhibitory concentration (IC_50_) values. They were calculated according to the protocol of Monks [40], where a dose-response curve was plotted for each compound and the concentration (IC_50_) that resulted in an inhibition of 50% was estimated through non-linear regression analysis.

#### 3.2.2. Antioxidant Activity 

##### Scavenging Activity on Free Radical DPPH

Free radical scavenging activity was measured using an adapted method of Mellors and Tappel [41]. The test was carried out on 96-well microplates. A 50 µL aliquot of the solution of the test compound was mixed with 150 µL of an ethanolic solution of DPPH (final concentration 100 µM). This mixture was incubated at 37 °C for 30 min, and the absorbance was then measured at 515 nm using an Ultra Microplate Reader (Synergy/HT, BIO-TEK Instrument, Inc., Winooski, VT, USA). The percentage of inhibition of each compound was determined by comparison with a 100 µM DPPH ethanolic blank solution.

##### Lipid Peroxidation Inhibition (TBARS)

The compounds **1**–**11** were evaluated employing the lipid peroxidation test (TBARS) in rat brain homogenate [31].

*Animals*: Adult male Wistar rats (200–250 g) were provided by Metropolitan Autonomous University-Xochimilco (UAM-X) animal facility. They were maintained at 25 °C on 12 light/12 h dark cycle with free access to food and water. The procedures with animals and their care were conducted in conformity with Mexican Official Norm for Animal Care and Handling (NOM-062-ZOO-1999; project ID: 124 “Evaluation of the antioxidant and antiinflammatory activities of curcumin and indazole derivatives”; date of approval: 28 May 2014; approved by the Intern Committee for the Use and Care of Laboratory Animals from UAM-X).

*The rat brain homogenate*: The animals were killed with CO_2_ and the whole brain was rapidly extracted, dissected, and homogenized into phosphate-buffer solution (PBS to 9.5 mM, pH = 7.4) to produce 10% (*w*/*v*) rat brain homogenate following Rossato’s et al. methodology [42].

The homogenate was centrifuged 10 min at 805 rpm and the resulting pellet was discarded. The protein content in the supernatant was determined according to the Lowry method [43]. Samples were adjusted to 2.66 mg/mL with PBS.

*Lipid peroxidation*: A mixture containing 375 µL of the supernatant and 50 µL of 10 µM EDTA was incubated 30 min at 37 °C in the presence of each compound-test. 50 µL of 100 µM FeSO_4_ solution was added to the mixture in order to obtain a final concentration of 10 µM and then each mixture was incubated at 37 °C for 60 min. TBARS was determined with slight modifications to Ohkawa´s methodology [44]. Then, 500 µL of thiobarbituric acid (TBA) solution (1% TBA in 0.05 N NaOH and 30% trichloroacetic acid in 1:1 proportion) was added to the mixture. After cooling on ice for 10 min, it was centrifuged at 12,879 rpm for 5 min and then incubated at 90 °C for 30 min. At room temperature, the absorbance of 200 µL of supernatant was measured at 540 nm in a Bio-Tek Microplate Reader Elx808. 1,1,3,3-tetramethoxypropane (TMP) was used as an external standard and the level of lipid peroxidation was expressed as nmol of malondialdehyde (MDA) per mg of protein.

Inhibition of the lipid test peroxidation was measured as percentage against a blank that did not contain any test compound. Quercetin and α-tocopherol were used as positive standards. The inhibition ratio (IR) was calculated using the following formula IR (%) = [(C − E)/C] × 100 where C represents the control of absorbance and E is the absorbance of the test sample. All data were represented as mean ± standard error of the mean (SEM). Data were analyzed by one-way ANOVA followed by Dunnett´s test for comparison against control. Values of *p* ≤ 0.05 (*) and *p* ≤ 0.01 (**) were considered statistically significant. Half maximal inhibitory concentration (IC_50_) values were estimated by means of a linear regression.

## 4. Conclusions

A series of 11 compounds were synthesized in this work. Five compounds, **1**–**5**, including curcumin and four derivatives were obtained in moderate yields employing an aldol condensation reaction. Additionally, six heterocyclic curcumin derivatives, **6**–**11**, were obtained in moderate to excellent yields. The cytotoxicity assays revealed that several compounds were more active than curcumin against HCT-15 and K562 cell lines. Mainly, **2** and **10** were the most active compounds against both cell lines. The results showed that most of the compounds have similar SAR against both cell lines. The SAR analyses suggest that a 3,4-dimethoxyphenyl substituent improves the cytotoxic activity. A change from a β-diketone group for a pyrazole or oxazole ring results in a gain in potency in most cases. Finally, the results of TBARS showed that **6** and **7** have strong antioxidant activity; particularly compound **7** is the best, even better than references curcumin, α-tocopherol, and quercetin.

## Figures and Tables

**Table 1:** Cytotoxic activity for compounds **1**–**11** in terms of percentage of growth inhibition against cancer cell lines.

Compound ^a^	U-251 MG ^d^	PC-3 ^e^	HCT-15 ^f^	K562 ^g^	SKLU-1 ^h^
**1** ^b^	59.1 ± 4.9	22.0 ± 2.4	81.2 ± 0.7	59.9 ± 5.0	72.4 ± 2.8
**2** ^c^	89.7 ± 4.0	78.0 ± 2.5	93.0 ± 2.3	93.0 ± 3.5	86.8 ± 2.4
**3**	41.1 ± 6.2	28.2 ± 1.4	33.8 ± 8.3	43.7 ± 6.2	24.2 ± 10.6
**4**	18.0 ± 3.5	26.3 ± 4.6	35.3 ± 11.9	27.8 ± 2.8	18.9 ± 2.1
**5** ^c^	73.7 ± 4.4	83.0 ± 6.2	94.1 ± 2.8	93.7 ± 5.8	91.4 ± 1.0
**6** ^c^	50.6 ± 5.4	55.8 ± 1.2	78.9 ± 7.0	84.5 ± 8.6	80.1 ± 10.8
**7** ^c^	63.3 ± 5.1	64.5 ± 1.7	77.0 ± 3.6	87.5 ± 10.9	88.6 ± 1.2
**8**	90.6 ± 5.1	91.5 ± 4.5	95.7 ± 4.2	97.3 ± 2.5	98.9 ± 1.0
**9**	15.2 ± 0.7	19.9 ± 0.7	33.5 ± 8.8	39.2 ± 5.7	5.2 ± 1.8
**10**	19.7 ± 4.2	63.4 ± 12.1	83.7 ± 7.4	71.2 ± 6.4	18.9 ± 5.1
**11**	34.0 ± 5.5	57.9 ± 3.7	69.3 ± 5.0	76.8 ± 3.5	51.2 ± 2.5

^a^ Data represents the mean of three or four independent replica measures ± SDE; ^b^ Tested at 12.5 µM due to its high activity at 50 µM; ^c^ Tested at 10 µM due to its high activity at 50 µM; ^d^ U-251 MG (glioblastoma); ^e^ PC-3 (human prostatic); ^f^ HCT-15 (human colorectal); ^g^ K562 (human chronic myelogenous leukemia); ^h^ SKLU-1 (non-small cell lung cancer).

**Table 2:** IC_50_ values (μM) for selected compounds (**1**, **2**, **5**–**8**, **10**, and **11**) against HCT-15 and K562 cell lines.

Compounds	IC_50_ µM	
	HCT-15	K562
**1**	13.9 ± 0.6	9.2 ± 0.4
**2**	2.3 ± 0.1	2.3 ± 0.3
**5**	4.8 ± 0.4	13.3 ± 0.6
**6**	5.8 ± 0.7	22.1 ± 0.9
**7**	6.2 ± 0.9	6.3 ± 0.6
**8**	5.0 ± 0.4	4.5 ± 0.2
**10**	2.8 ± 0.1	3.0 ± 0.4
**11**	12.7 ± 0.1	8.8 ± 0.3
Adriamycin	0.0050 ± 0.0009	0.0140 ± 0.0009

Data shown is the mean ± standard error from three independent experiments. IC_50_: half maximal inhibitory concentration.

**Table 3:** Antioxidant activity of selected compound.

Compound	IC_50_ (µM) TBARS ^a^	IC_50_ (µM) DPPH ^b^
**1**	3.22 ± 0.32	23.52 ± 1.20
**2**	18.67 ± 2.28	Inactive
**6**	1.31 ± 0.15	25.92 ± 1.73
**7**	0.63 ± 0.02	25.81 ± 2.97
**10**	15.30 ± 0.46	Inactive
Quercetin	1.49 ± 0.03	10.89 ± 0.47
α-Tocopherol	6.78 ± 2.16	31.74 ± 1.04

^a^ TBARS: thiobarbituric acid reactive substances assay; ^b^ DPPH: 2,2-Diphenyl-1-picrylhydrazyl assay.

## Supplementary Materials

Supplementary materials are available online.
