# Supplementary material for: Synthesis of Curcuminoids and Evaluation of Their Cytotoxic and Antioxidant Properties
Source: Molecules. 2017 Apr 14;22(4):633. doi: 10.3390/molecules22040633 (PMC6154528; doi:10.3390/molecules22040633)
Supplement: Supplementary file 1 [file molecules-22-00633-s001.pdf]

Supplementary materials

Synthesis of curcuminoids and evaluation of their cytotoxic and antioxidant properties

María Concepción Lozada-García[1], † Raúl G. Enríquez[1], Teresa O. Ramírez-Apán[2], Antonio Nieto-Camacho[2] , Juan F. Palacios-Espinosa[1], Zeltzin Custodio-Galván[1], Olivia Soria-Arteche[1*] and Jaime Pérez-Villanueva[1*]

[1]Departamento de Sistemas Biológicos, División de Ciencias Biológicas y de la Salud, Universidad Autónoma Metropolitana-Xochimilco (UAM-X), Ciudad de México 04960, México

[2]Instituto de Química, Universidad Nacional Autónoma de México (UNAM), Ciudad de México 04510, México

* These authors contributed equally to this work

Correspondence: jpvillanueva@correo.xoc.uam.mx. (J.P-V.); soriao@correo.xoc.uam.mx (O.S-A.); Tel: +52 5483 7259 (J.P-V. and O.S-A.).

Table of contents

Figure	pages
S1. Mass spectrum of compound 1 recorded in a MStation equipment by direct inlet and EI mode ion detection	3
S2. NMR ^{1}H spectrum of compound **1** recorded in a Bruker 500 MHz and $CDCl_3$ as solvent.	3
S3. NMR ^{13}C spectrum of compound **1** recorded in a Bruker 500 MHz and $CDCl_3$ as solvent.	4
S4. Mass spectrum of compound **2** recorded in a Jeol JMS-AX505HA equipment by direct inlet and EI mode ion detection.	5
S5. NMR ^{1}H spectrum of compound **2** recorded in a Gemini 200 MHz of Varian and $CDCl_3$ as solvent.	5
S6. NMR ^{13}C spectrum of compound **2** recorded in a Gemini 200 MHz of Varian and $CDCl_3$ as solvent.	6
S7. NMR ^{1}H spectrum of compound **3** recorded in a Bruker 500 MHz and $CDCl_3$ as solvent.	7
S8. NMR ^{13}C spectrum of compound **3** recorded in a Bruker 500 MHz and $CDCl_3$ as solvent.	7

S9. Mass spectrum of compound **4** recorded in a Jeol JMS-AX505HA equipment by direct inlet and EI mode ion detection.	8
S10. NMR ^{1}H spectrum of compound **4** recorded in a Bruker 500 MHz and $CDCl_3$ as solvent.	8
S11. NMR ^{13}C spectrum of compound **4** recorded in a Bruker 500 MHz and $CDCl_3$ as solvent.	9
S12. Mass spectrum of compound **5** recorded in a Jeol JMS-AX505HA equipment by direct inlet and EI mode ion detection.	10
S13. NMR ^{1}H spectrum of compound **5** recorded in a Varian 600 MHz and DMSO as solvent.	10
S14. NMR ^{13}C spectrum of compound **5** recorded in a Varian 600 MHz and DMSO as solvent.	11
S15. Mass spectrum of compound **6** in a JEOL GCmate equipment by direct inlet and EI mode ion detection.	12
S16. NMR ^{1}H spectrum of compound **6** recorded in a Varian 600 MHz and $CDCl_3$ as a solvent.	12
S17. NMR ^{13}C spectrum of compound **6** recorded in a Varian 600 MHz and $CDCl_3$ as solvent.	13
S18. Mass spectrum of compound **7** in a JEOL GCmate equipment by direct inlet and EI mode ion detection.	14
S19. NMR ^{1}H spectrum of compound **7** recorded in a Varian 600 MHz and $CDCl_3$ as solvent.	14
S20. NMR ^{13}C spectrum of compound **7** recorded in a Varian 600 MHz and $CDCl_3$ as solvent.	15
S21. Mass spectrum of compound **8** recorded in an MStation equipment by direct inlet and EI mode ion detection.	16
S22. NMR ^{1}H spectrum of compound **8** recorded in a Varian 600 MHz and $CDCl_3$ as solvent.	16
S23. NMR ^{13}C spectrum of compound **8** recorded in a Varian 600 MHz and $CDCl_3$ as solvent.	17
S24. Mass spectrum of compound **9** recorded in a micrOTOF equipment and ESI mode ion detection.	18
S25. NMR ^{1}H spectrum of compound **9** recorded in a Varian 600 MHz and $CDCl_3$ as solvent.	18
S26. NMR ^{13}C spectrum of compound **9** recorded in a Varian 600 MHz and $CDCl_3$ as solvent.	19
S27. Mass spectrum of compound **10** recorded in a Jeol JMS-AX505HA equipment by direct inlet and EI mode ion detection.	20
S28. NMR ^{1}H spectrum of compound **10** recorded in a Bruker 300 MHz and $CDCl_3$ as solvent.	20
S29. NMR ^{13}C spectrum of compound **10** recorded in a Bruker 300 MHz and $CDCl_3$ as solvent.	21
S30. Mass spectrum of compound **11** recorded in a Jeol JMS-AX505HA equipment by direct inlet and EI mode ion detection.	22
S31. NMR ^{1}H spectrum of compound **11** recorded in a Bruker 300 MHz and $CDCl_3$ as solvent.	22
S32. NMR ^{13}C spectrum of compound **11** recorded in a Bruker 300 MHz and $CDCl_3$ as solvent.	23

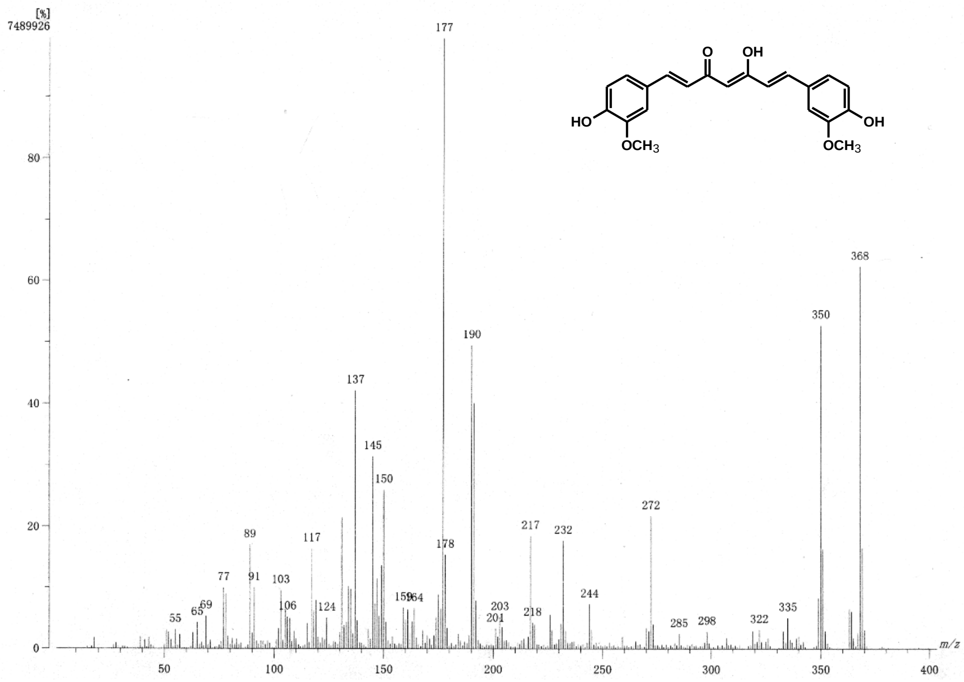

S1. Mass spectrum of compound 1 recorded in a MStation equipment by direct inlet and EI mode ion detection.

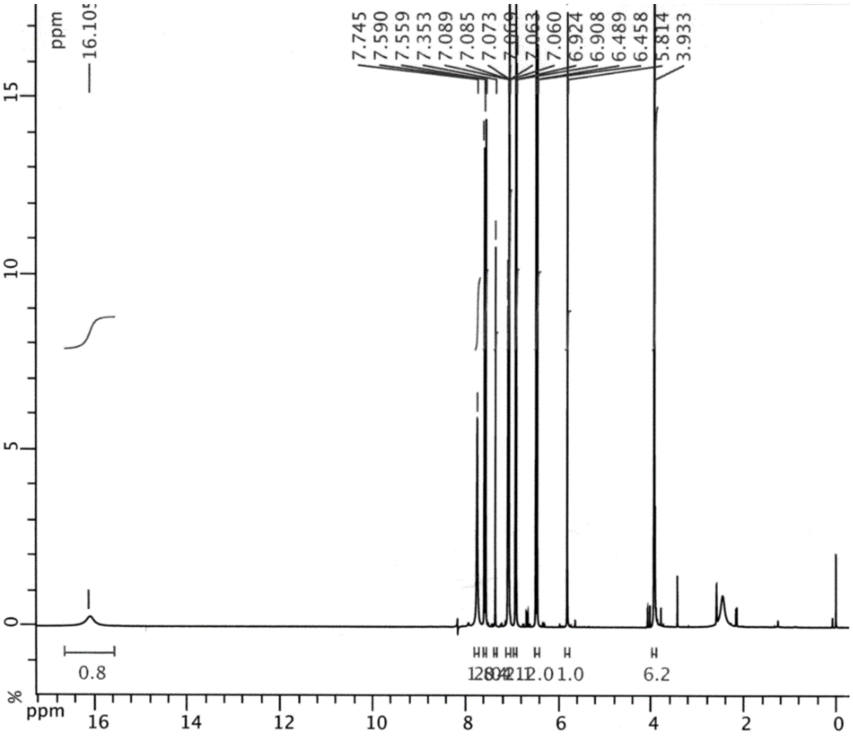

S2. NMR ^{1}H spectrum of compound **1** recorded in a Bruker 500 MHz and $CDCl_3$ as solvent.

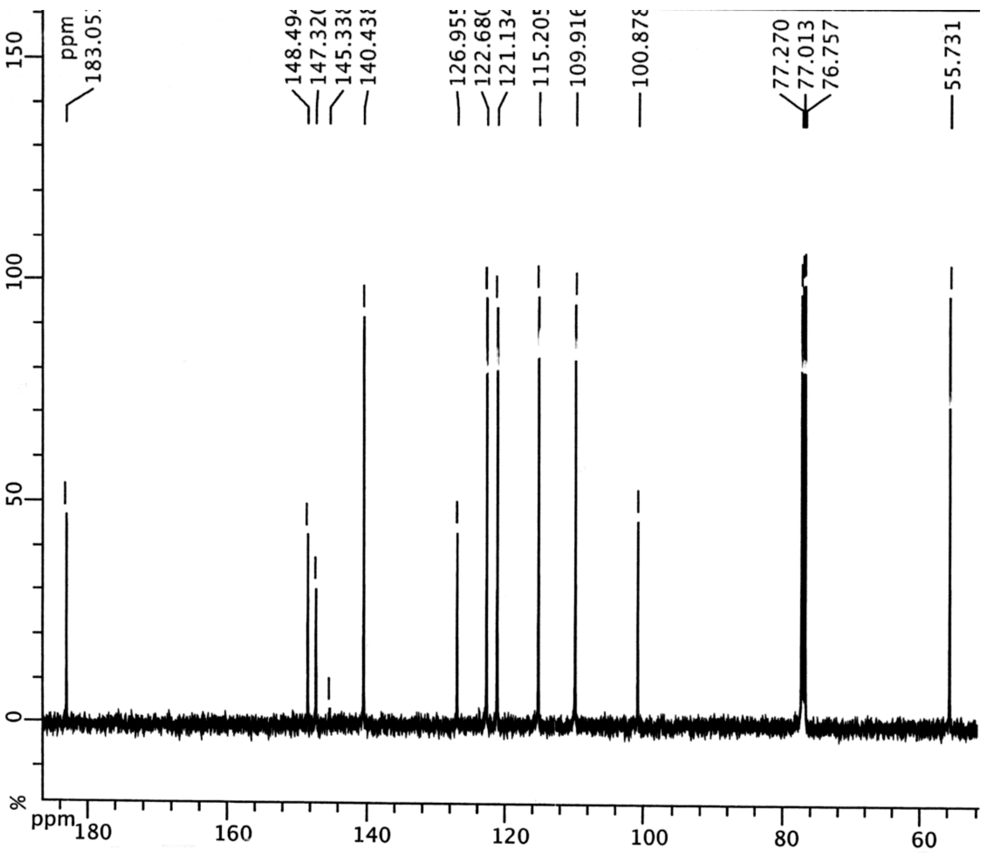

S3. NMR ^{13}C spectrum of compound **1** recorded in a Bruker 500 MHz and $CDCl_3$ as solvent.

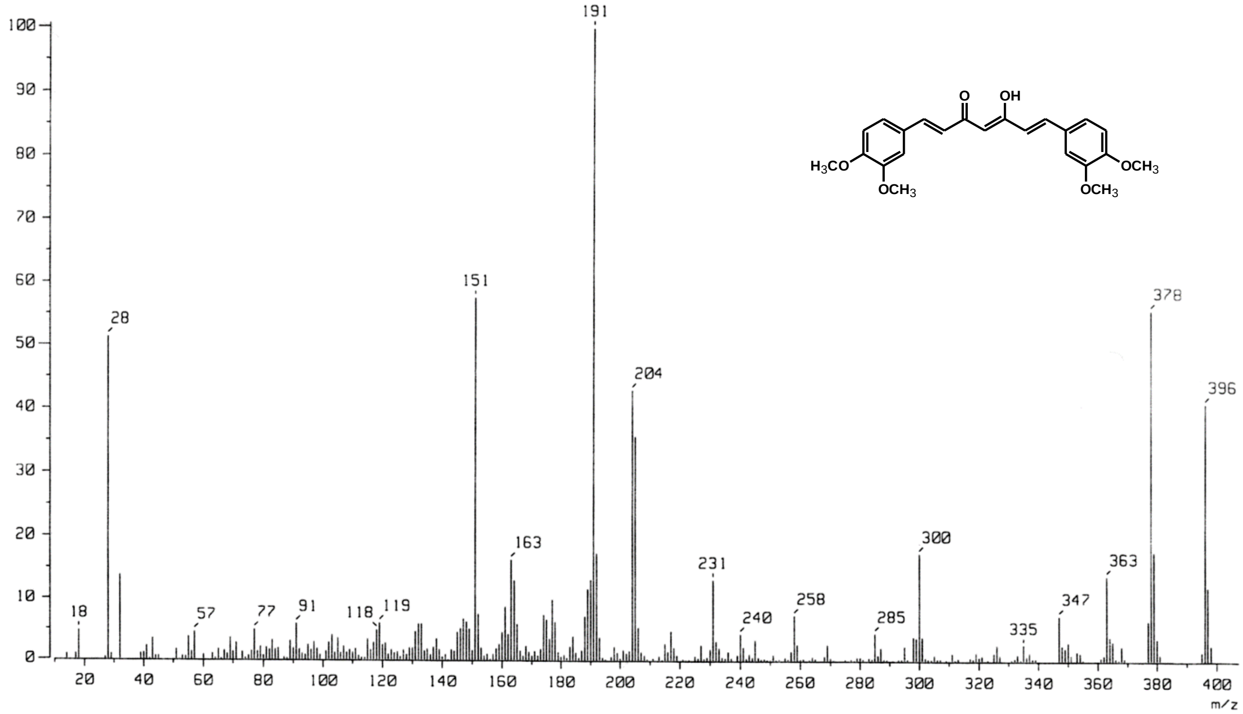

S4. Mass spectrum of compound **2** recorded in a Jeol JMS-AX505HA equipment by direct inlet and EI mode ion detection.

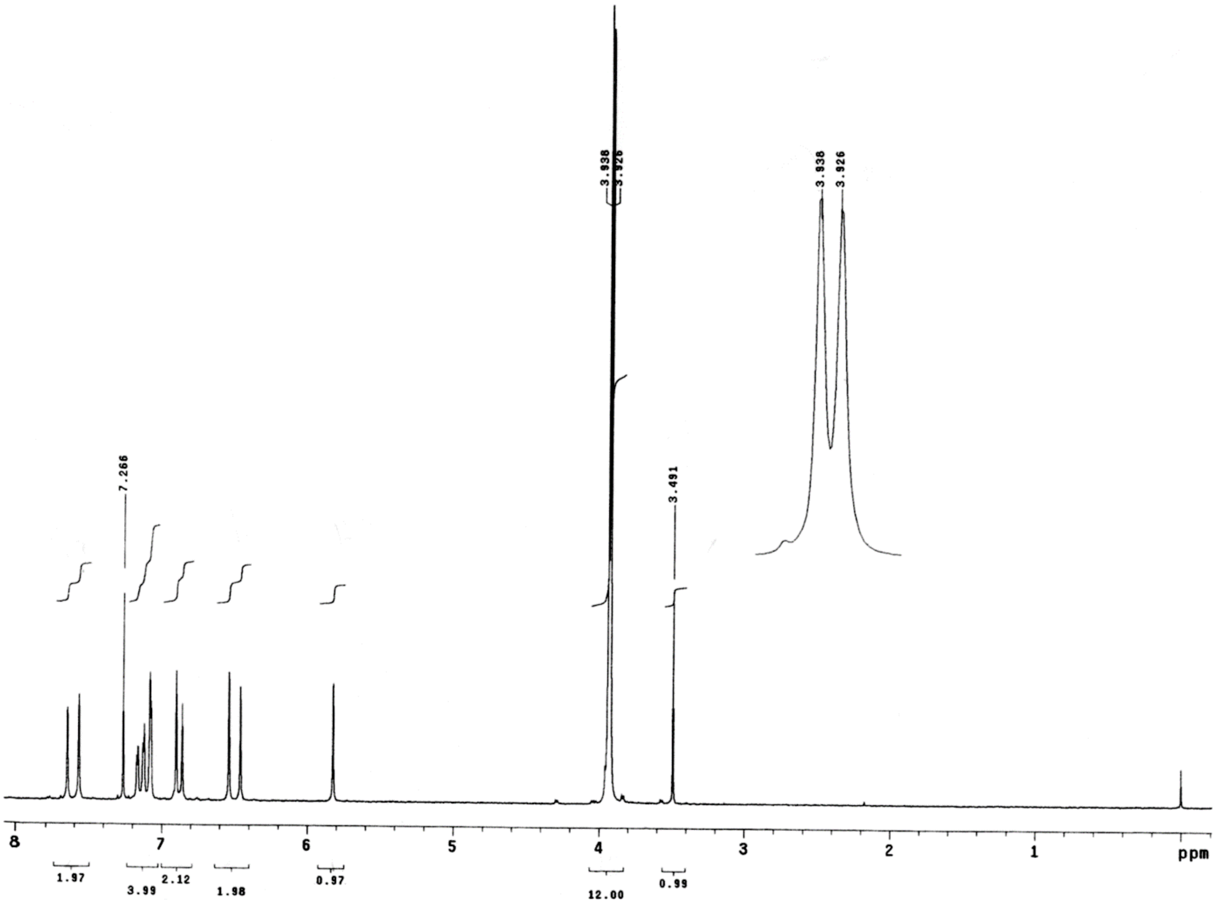

S5. NMR ^{1}H spectrum of compound **2** recorded in a Gemini 200 MHz of Varian and $CDCl_3$ as solvent.

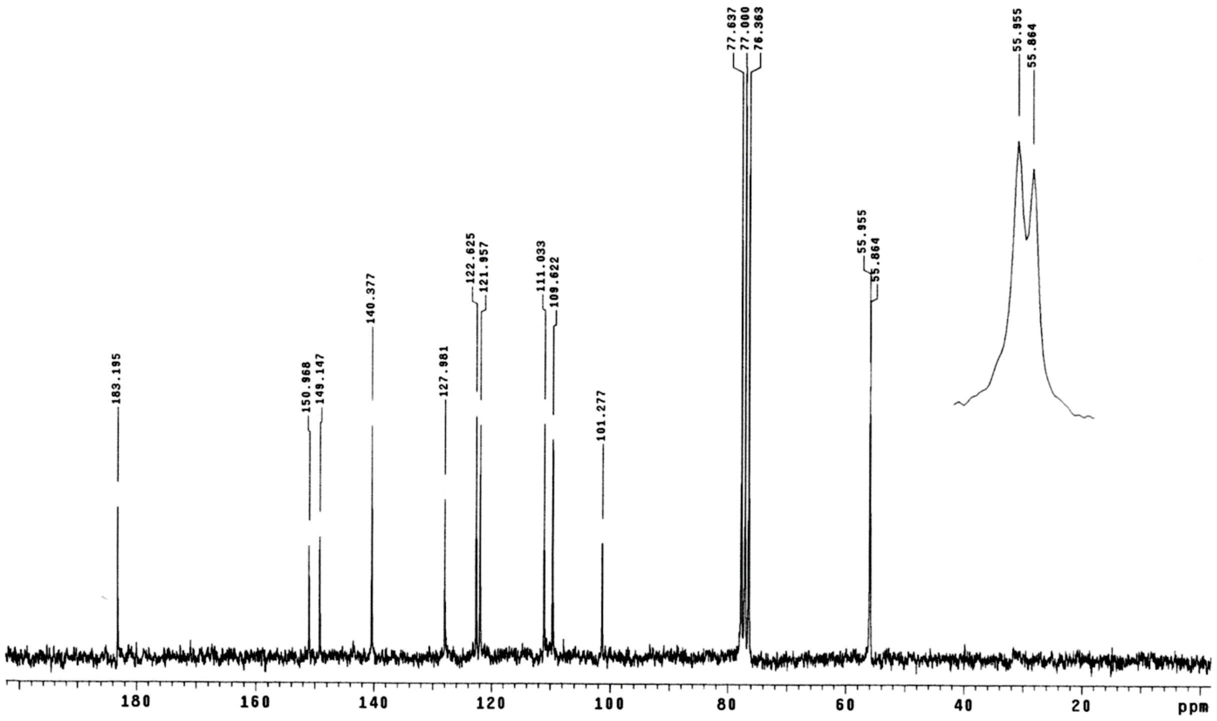

S6. NMR ^{13}C spectrum of compound **2** recorded in a Gemini 200 MHz of Varian and $CDCl_3$ as solvent.

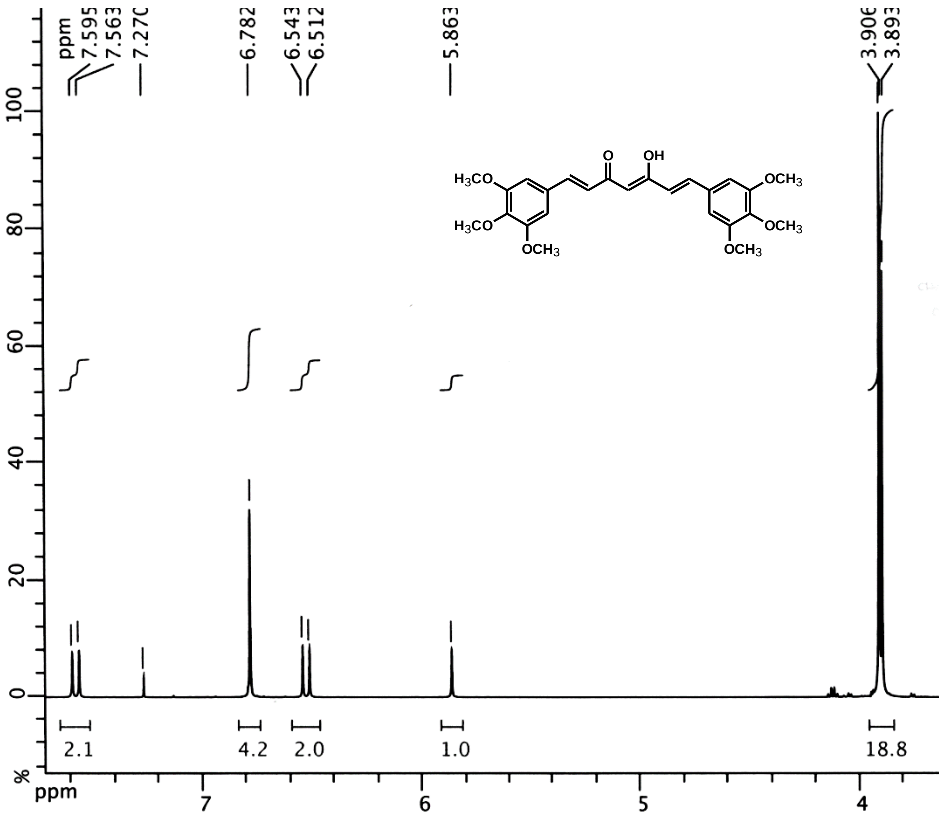

S7. NMR ^{1}H spectrum of compound **3** recorded in a Bruker 500 MHz and $CDCl_3$ as solvent.

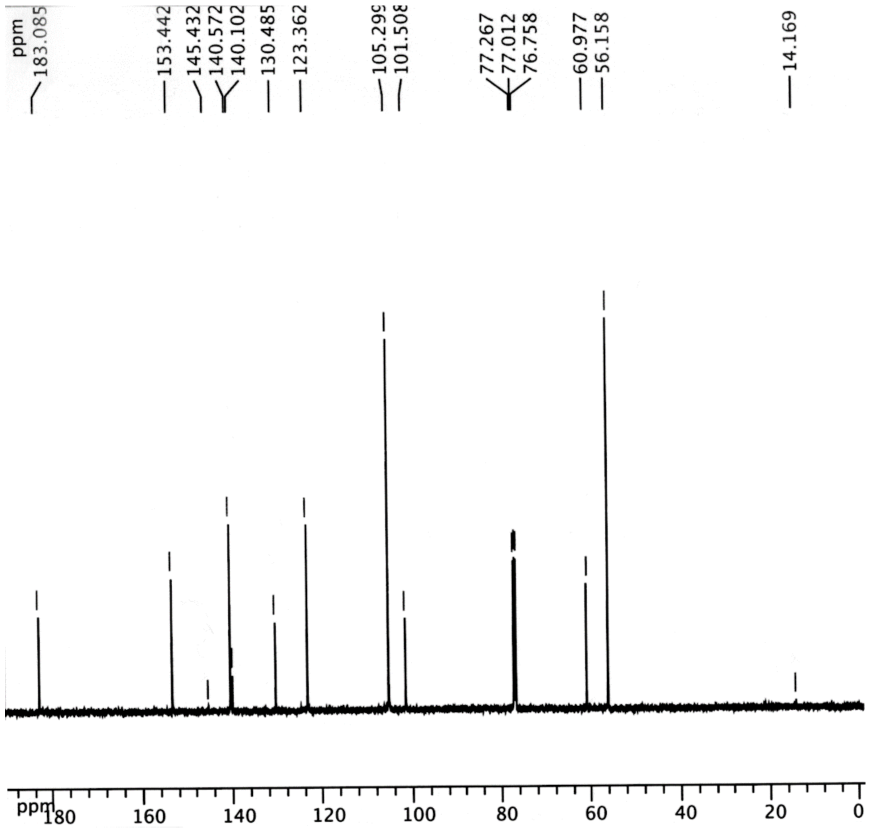

S8. NMR ^{13}C spectrum of compound **3** recorded in a Bruker 500 MHz and $CDCl_3$ as solvent.

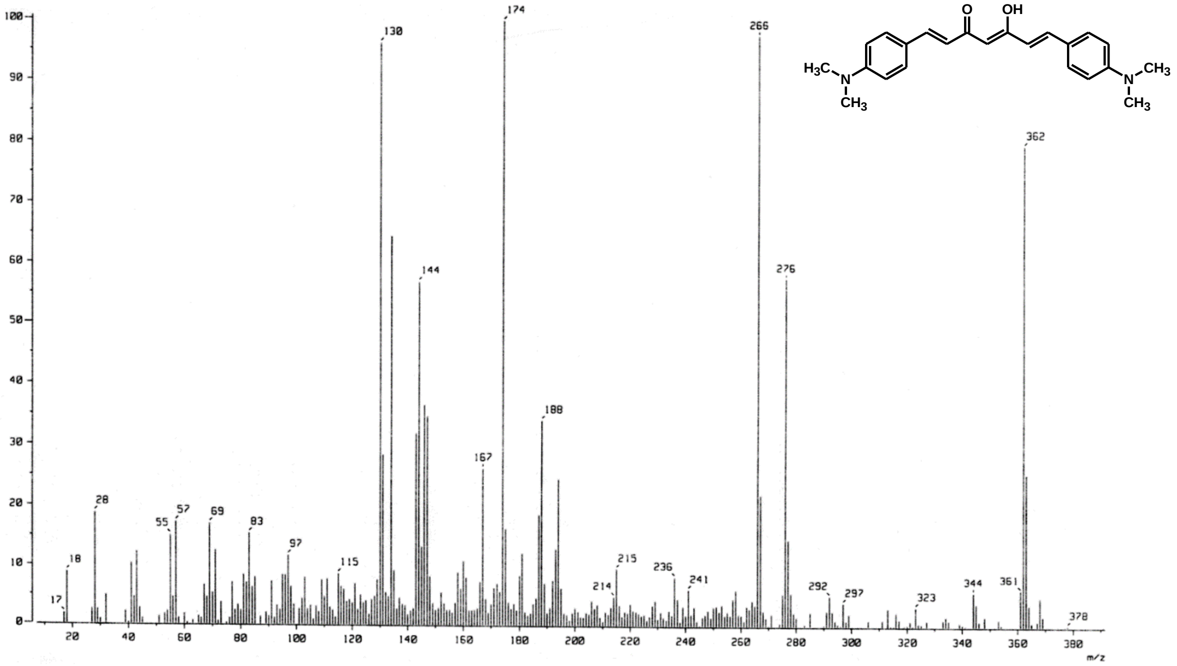

S9. Mass spectrum of compound **4** recorded in a Jeol JMS-AX505HA equipment by direct inlet and EI mode ion detection.

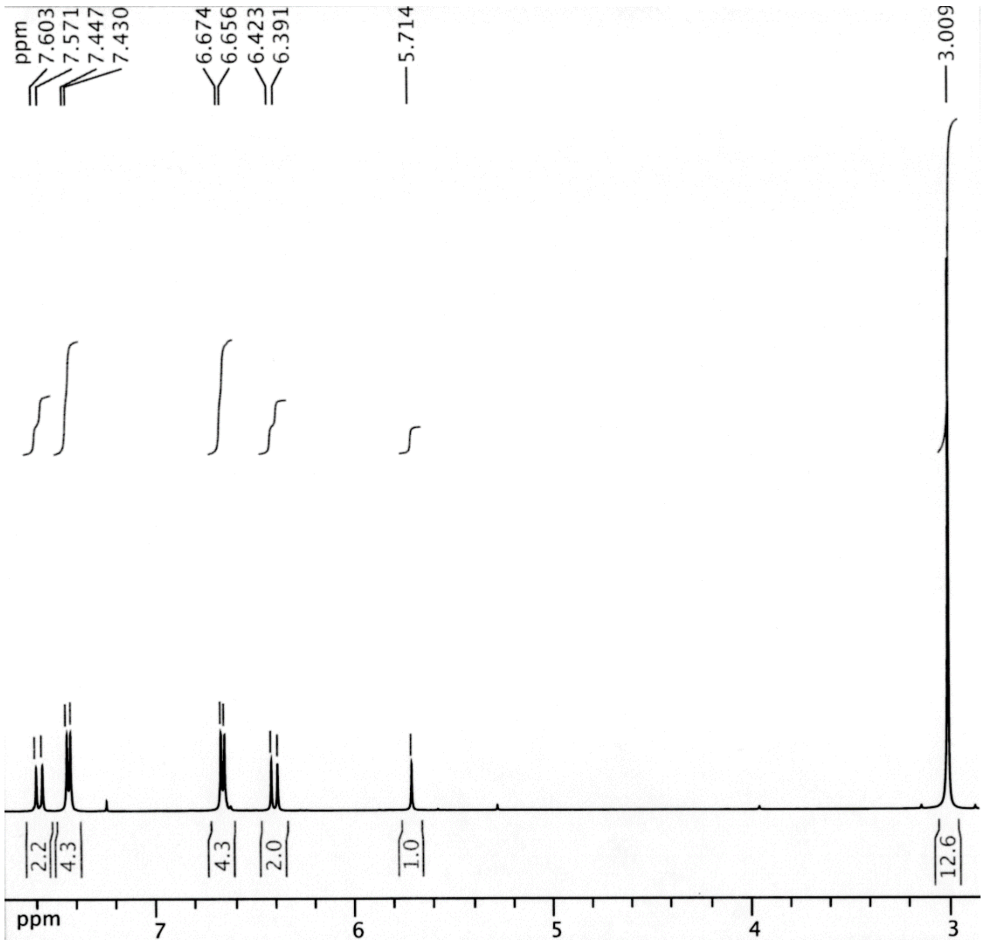

S10. NMR ^{1}H spectrum of compound **4** recorded in a Bruker 500 MHz and $CDCl_3$ as solvent.

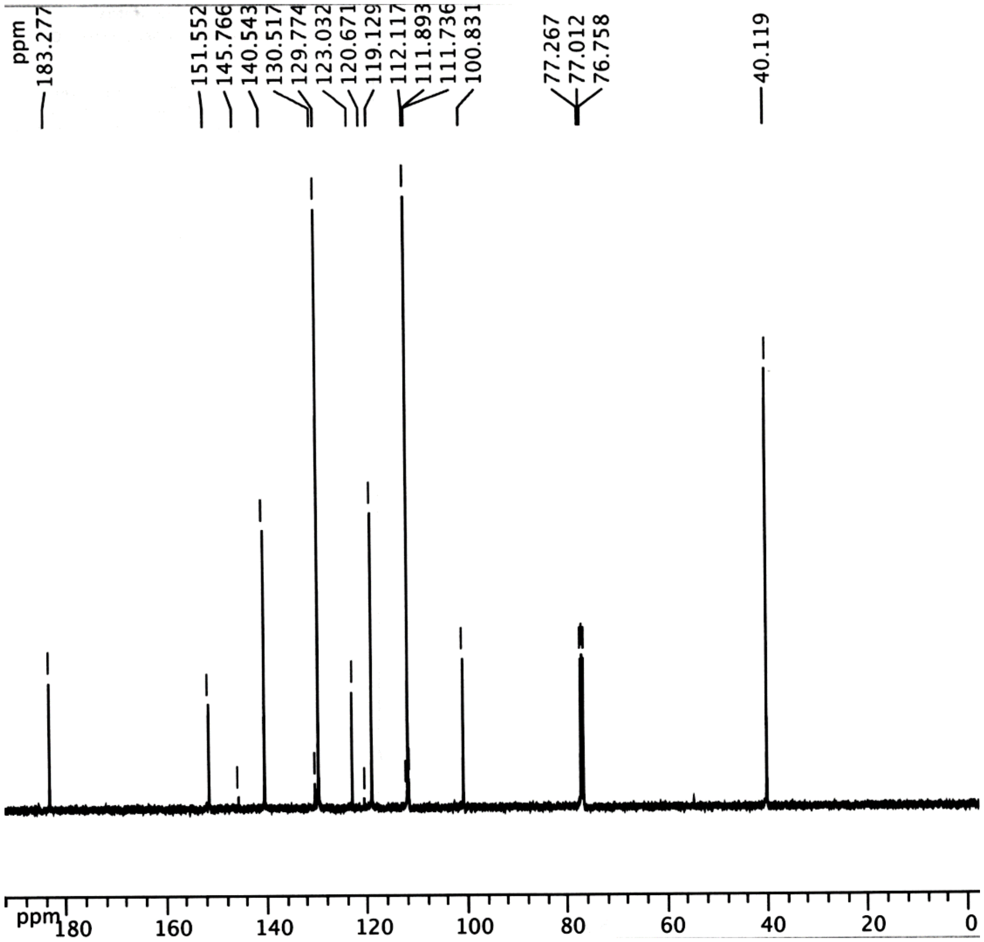

S11. NMR ^{13}C spectrum of compound **4** recorded in a Bruker 500 MHz and $CDCl_3$ as solvent.

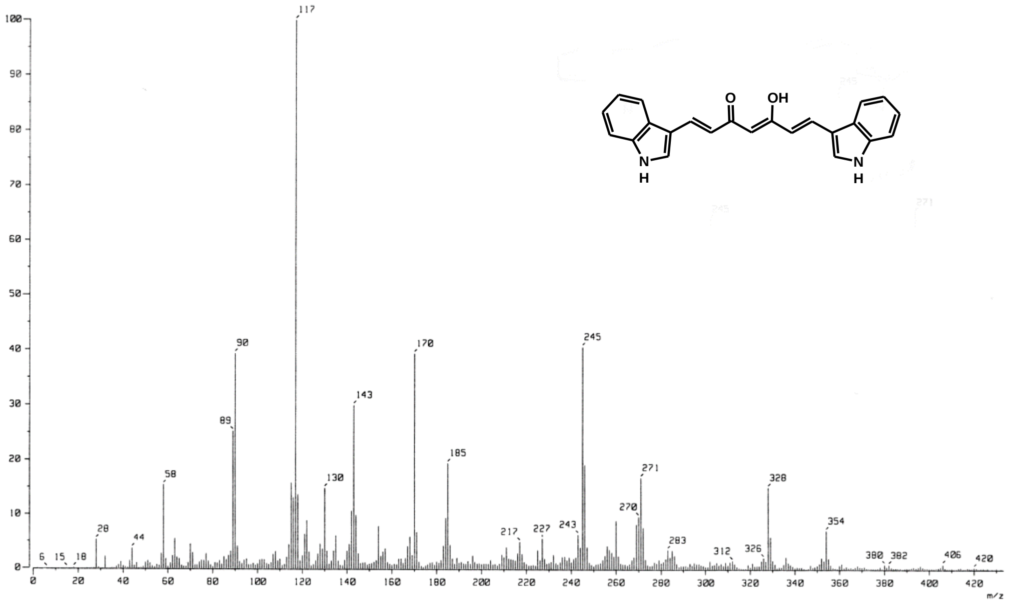

S12. Mass spectrum of compound **5** recorded in a Jeol JMS-AX505HA equipment by direct inlet and EI mode ion detection.

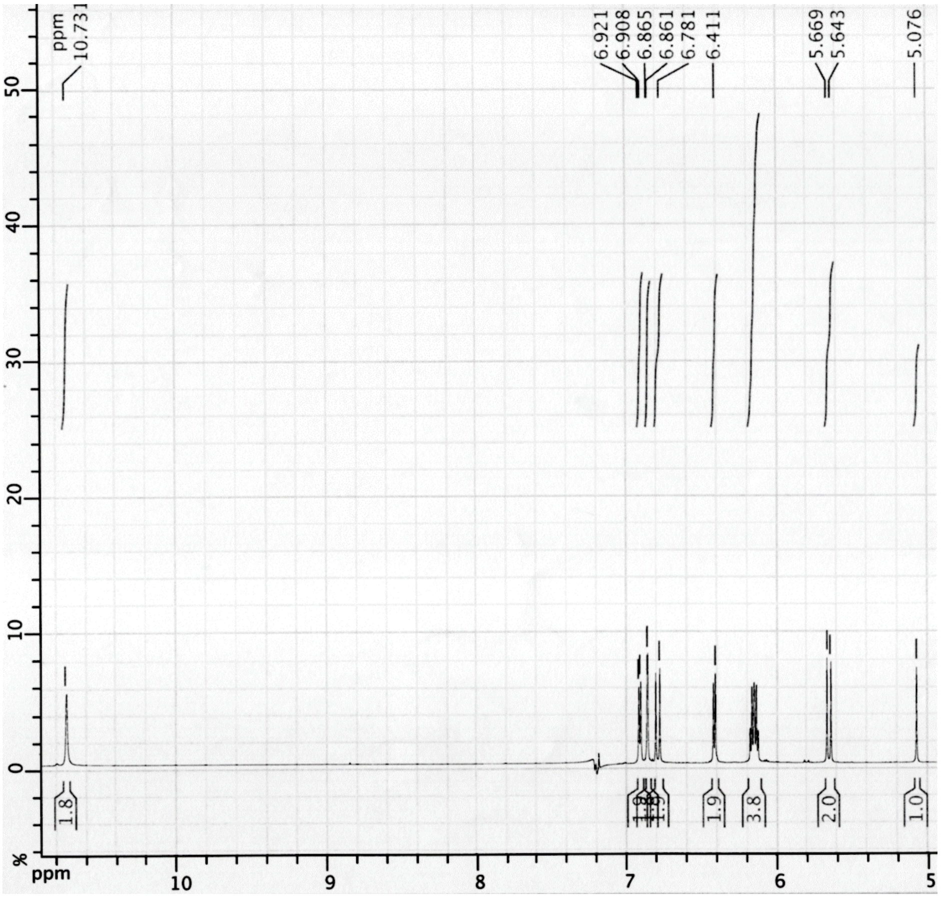

S13. NMR ^{1}H spectrum of compound **5** recorded in a Varian 600 MHz and DMSO as solvent.

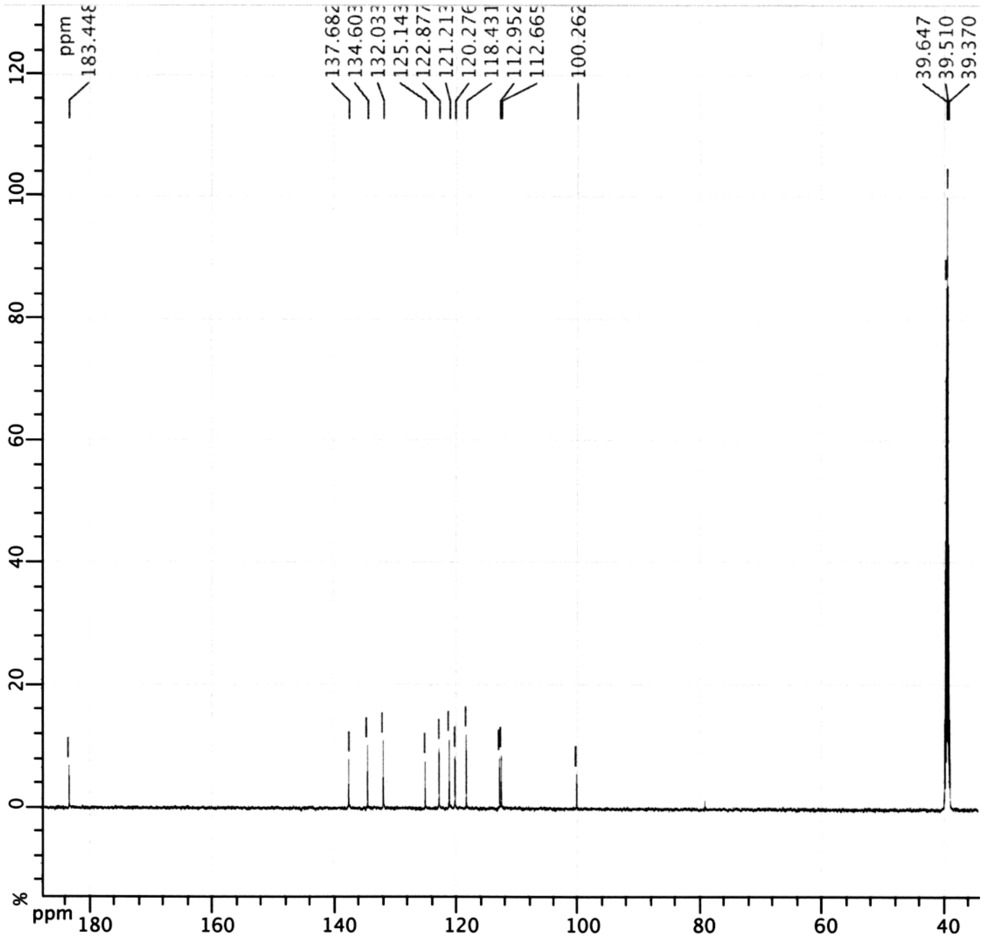

S14. NMR ^{13}C spectrum of compound **5** recorded in a Varian 600 MHz and DMSO as solvent.

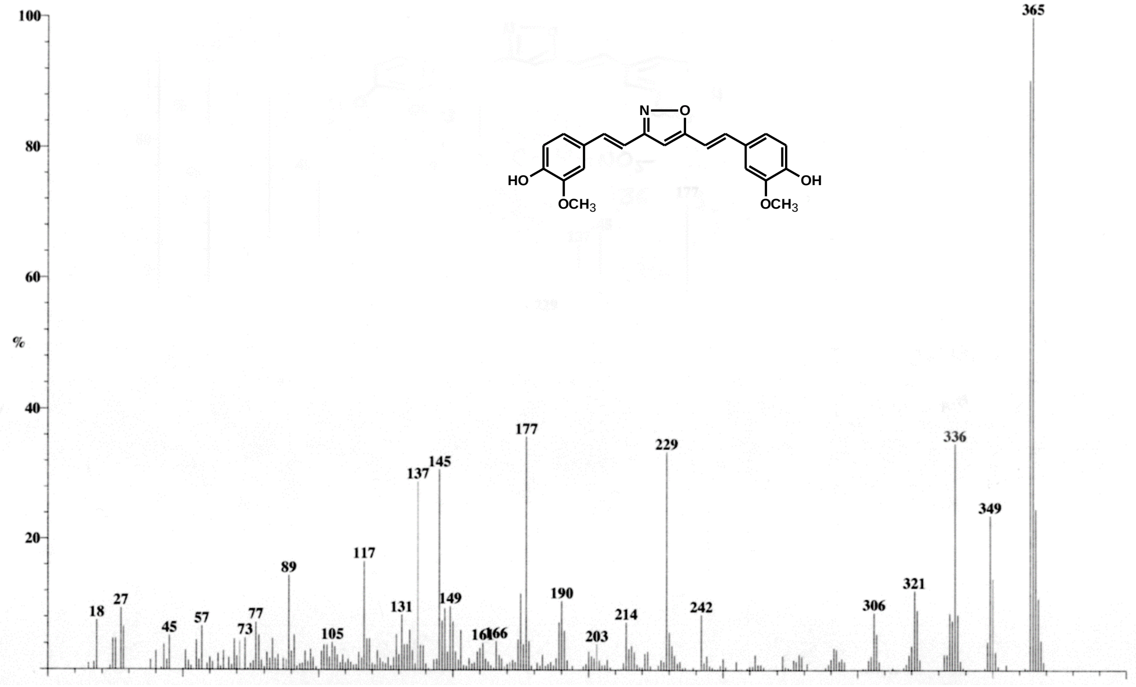

S15. Mass spectrum of compound **6** in a JEOL GCmate equipment by direct inlet and EI mode ion detection.

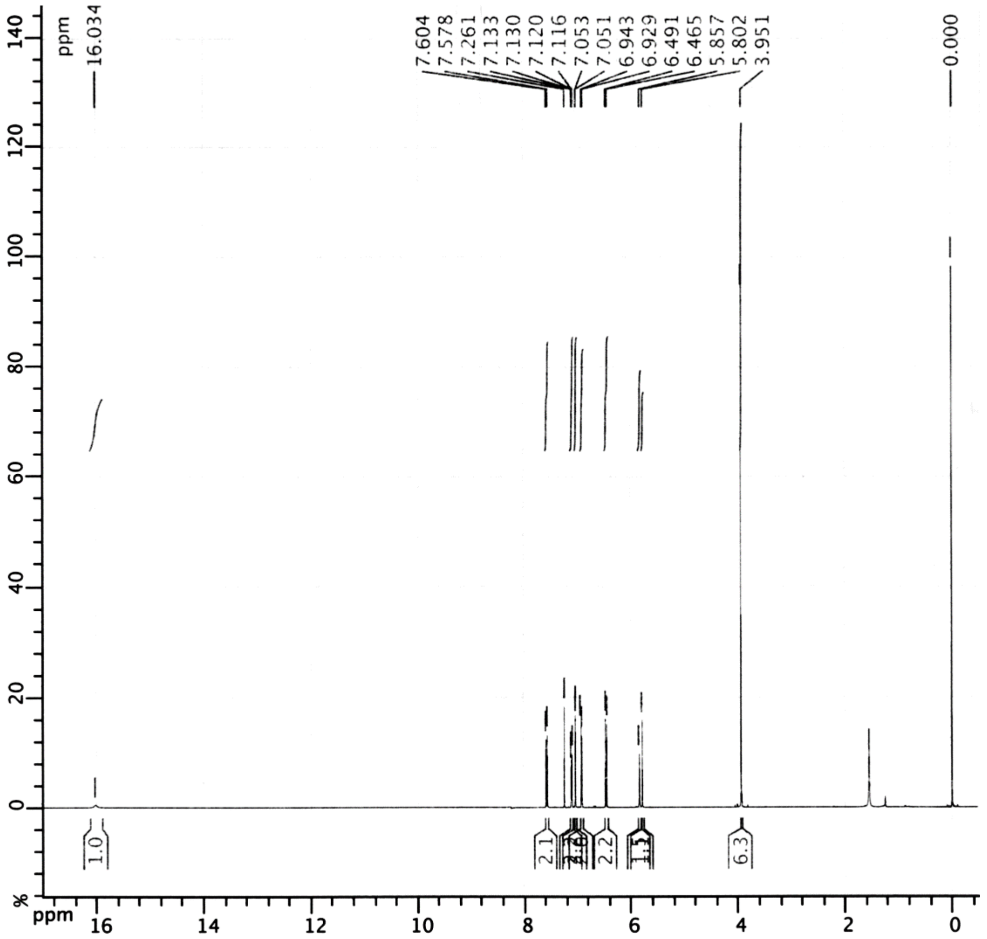

S16. NMR ^1H spectrum of compound **6** recorded in a Varian 600 MHz and $CDCl_3$ as a solvent.

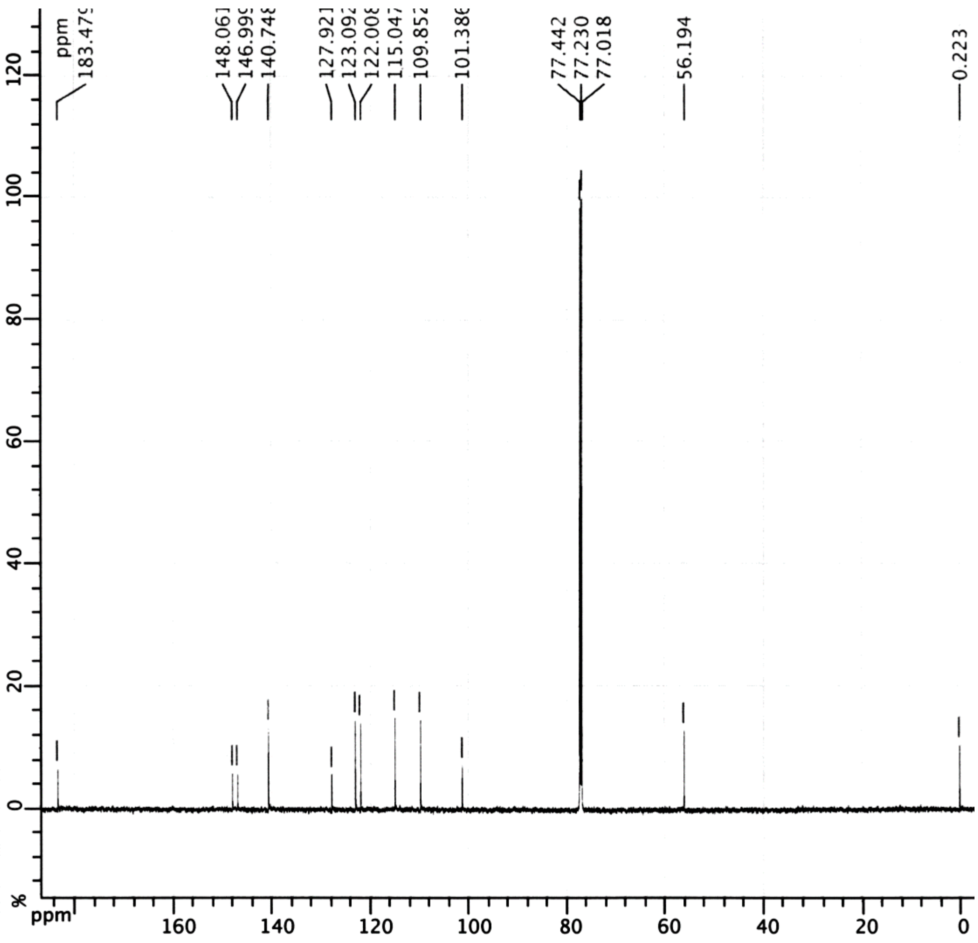

S17. NMR ^{13}C spectrum of compound **6** recorded in a Varian 600 MHz and $CDCl_3$ as solvent.

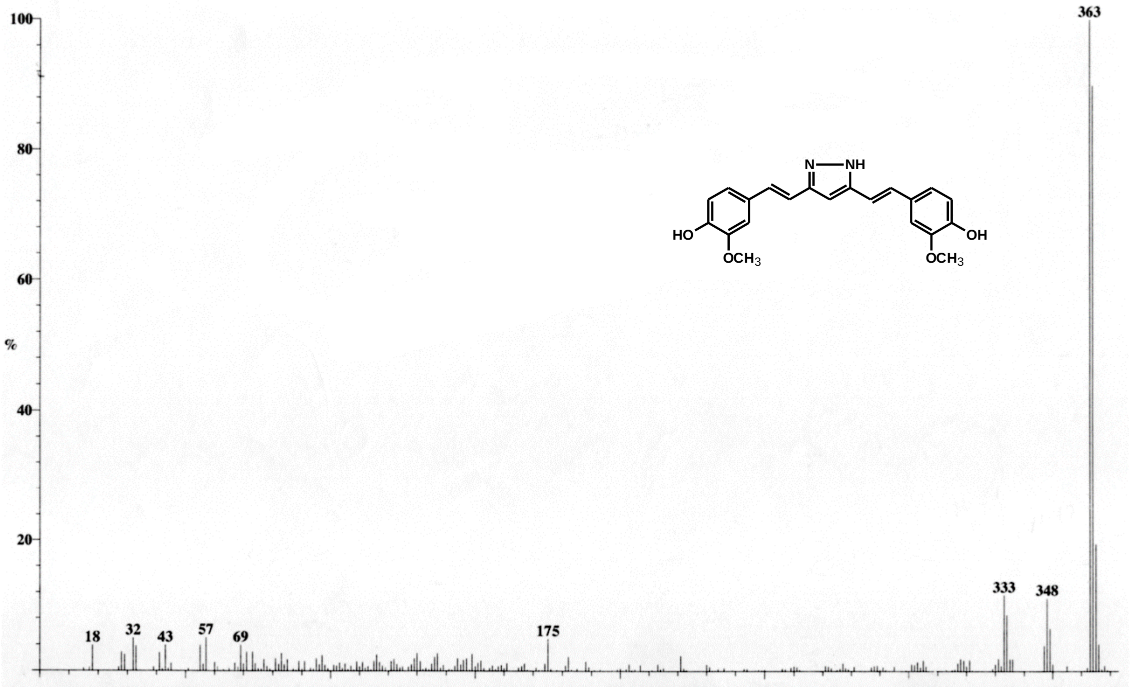

S18. Mass spectrum of compound **7** in a JEOL GCmate equipment by direct inlet and EI mode ion detection.

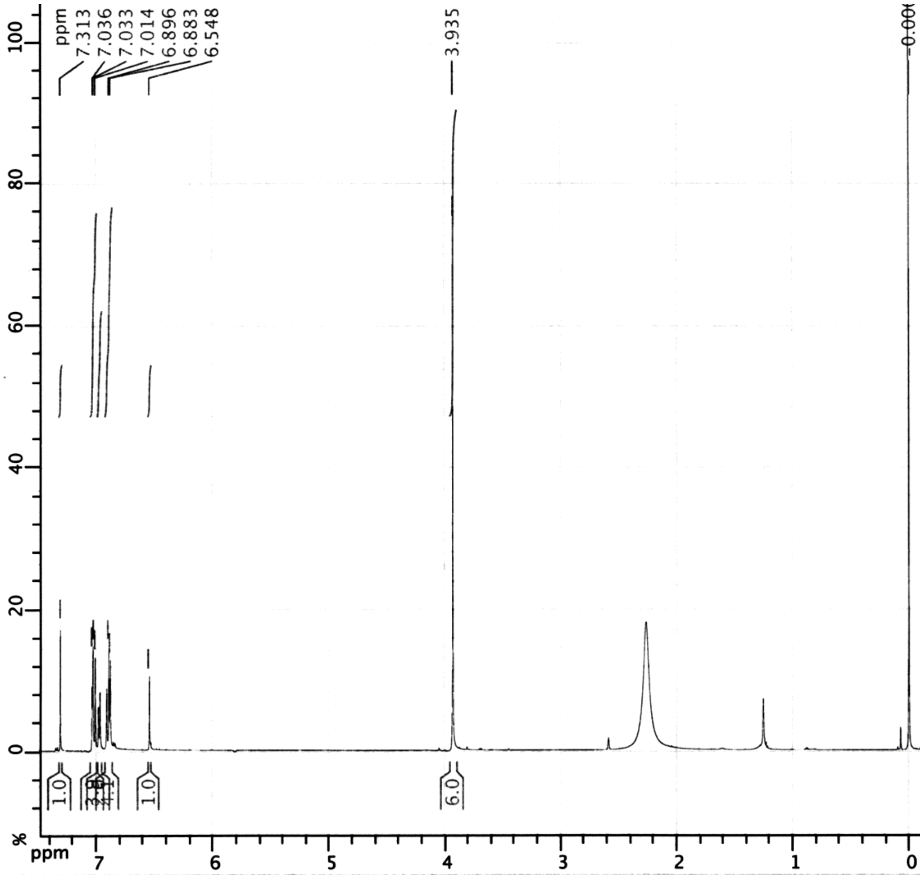

S19. NMR ^{1}H spectrum of compound **7** recorded in a Varian 600 MHz and $CDCl_3$ as solvent.

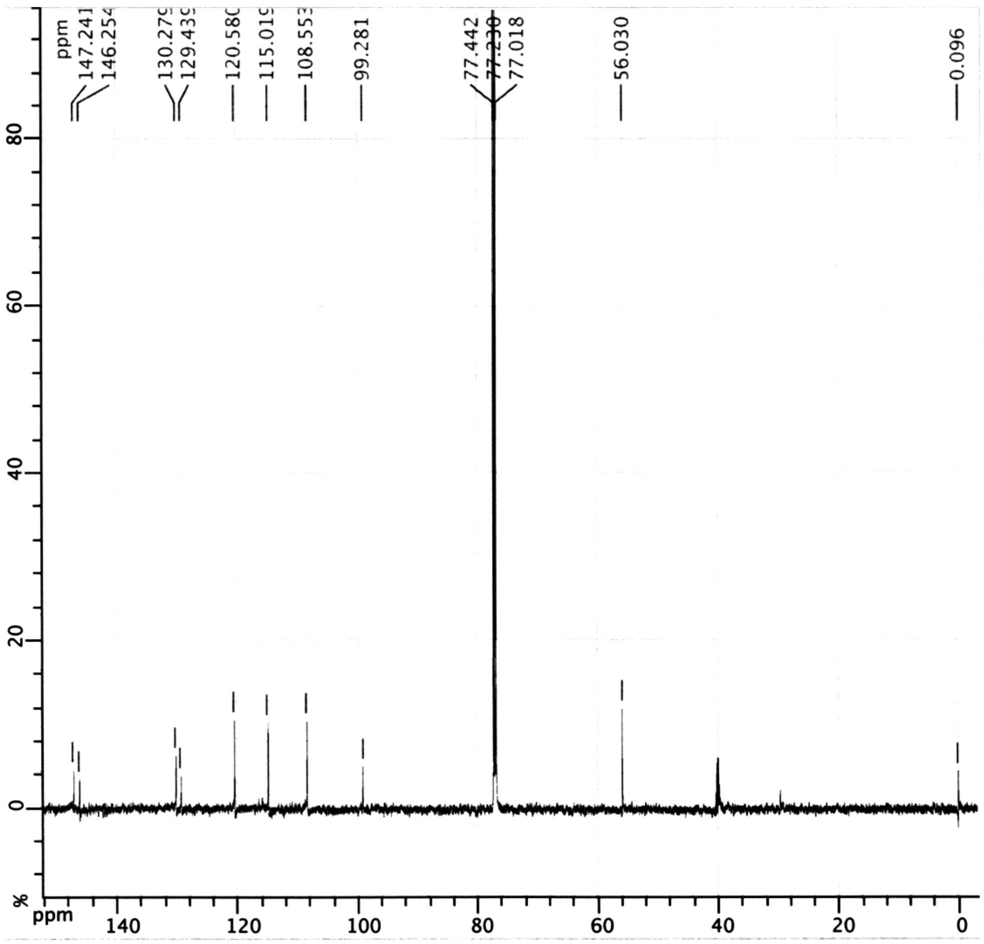

S20. NMR ^{13}C spectrum of compound **7** recorded in a Varian 600 MHz and $CDCl_3$ as solvent.

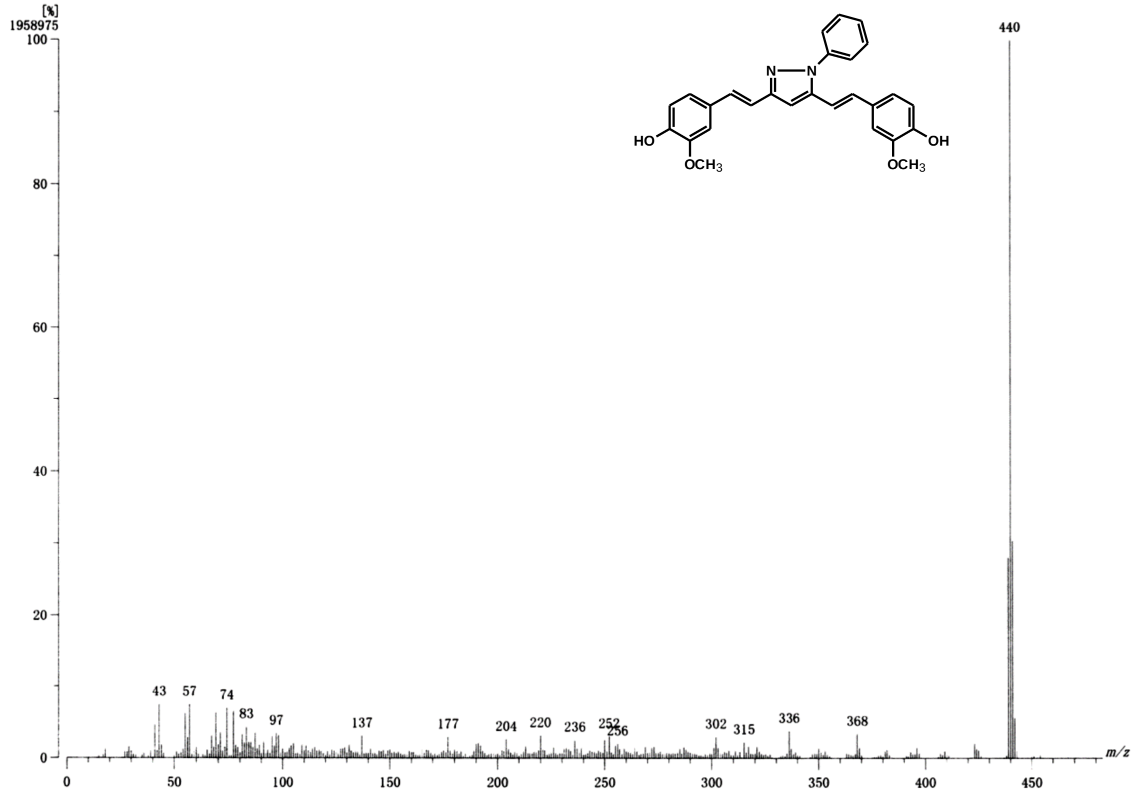

S21. Mass spectrum of compound **8** recorded in an MStation equipment by direct inlet and EI mode ion detection.

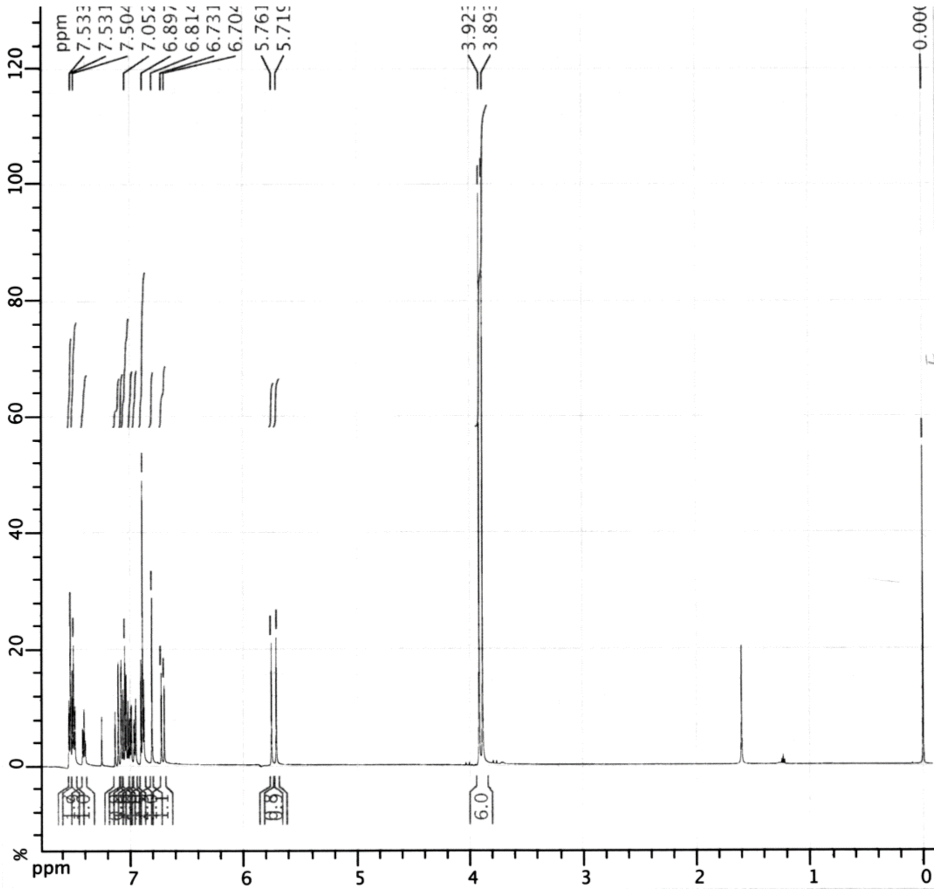

S22. NMR ^{1}H spectrum of compound **8** recorded in a Varian 600 MHz and $CDCl_3$ as solvent.

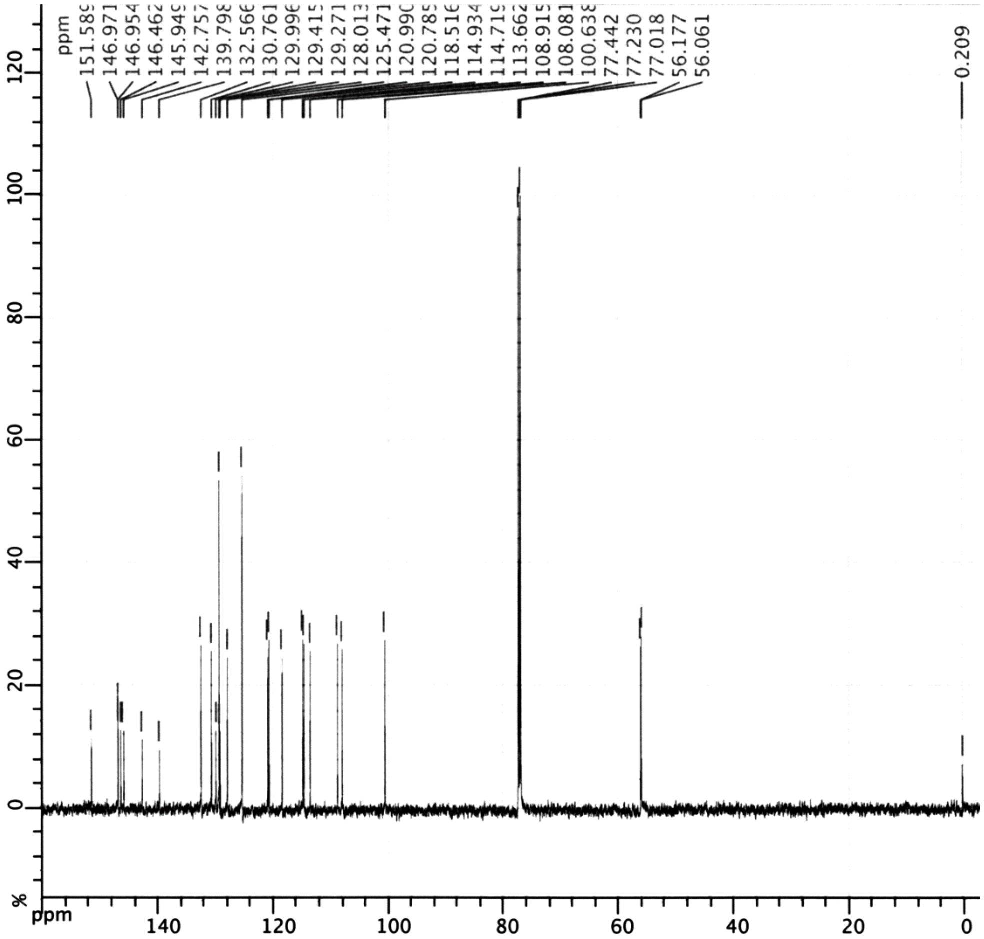

S23. NMR ^{13}C spectrum of compound **8** recorded in a Varian 600 MHz and $CDCl_3$ as solvent.

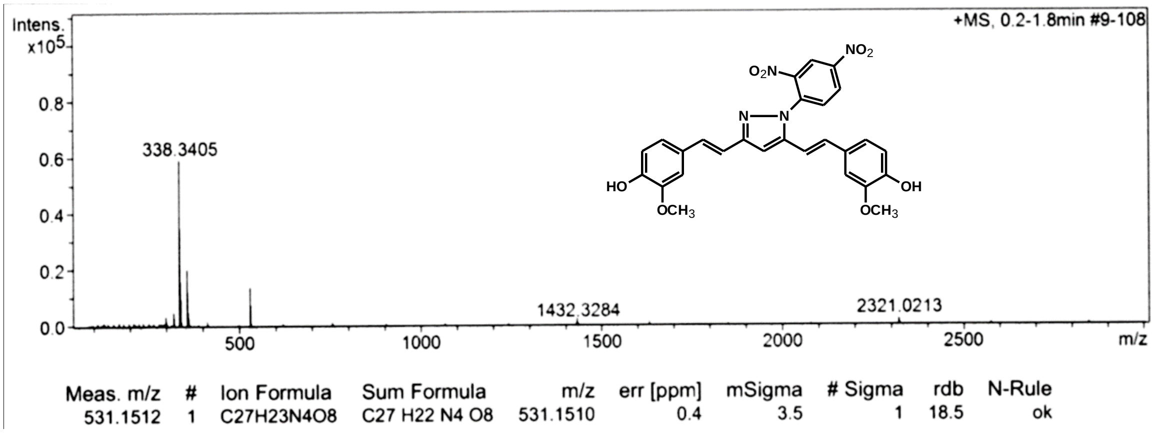

S24. Mass spectrum of compound **9** recorded in a micrOTOF equipment and ESI mode ion detection.

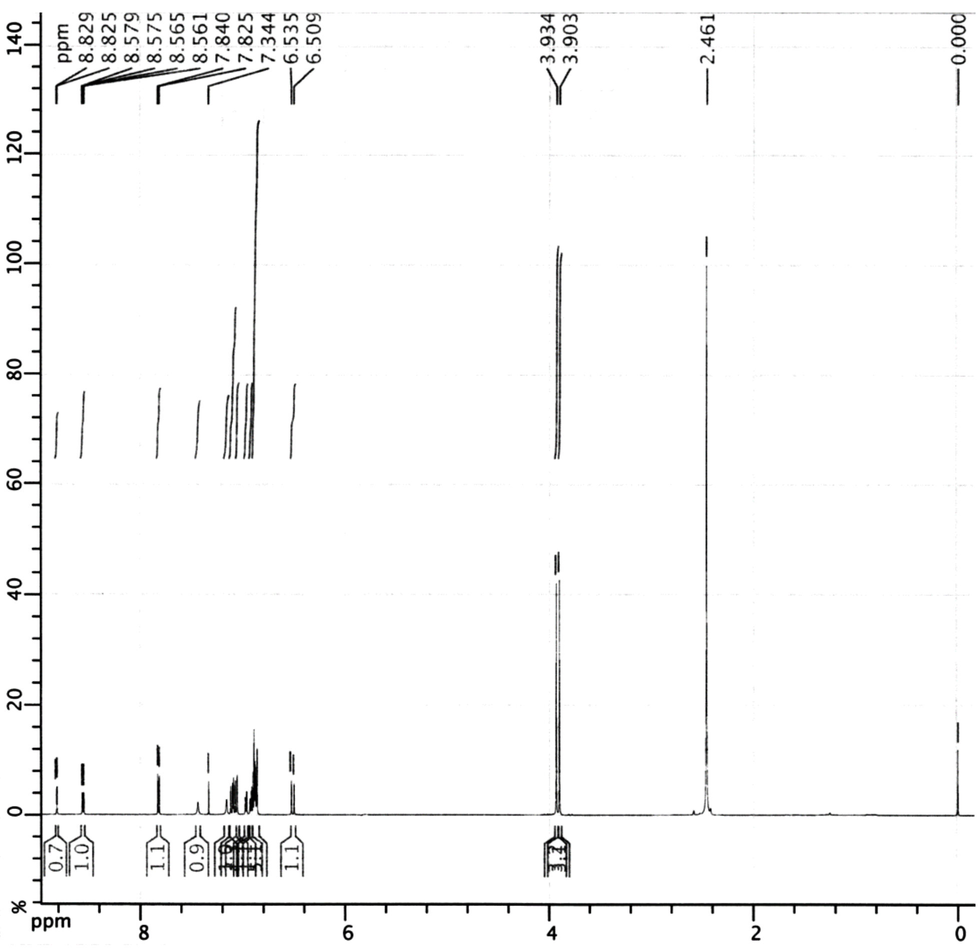

S25. NMR ^{1}H spectrum of compound **9** recorded in a Varian 600 MHz and $CDCl_3$ as solvent.

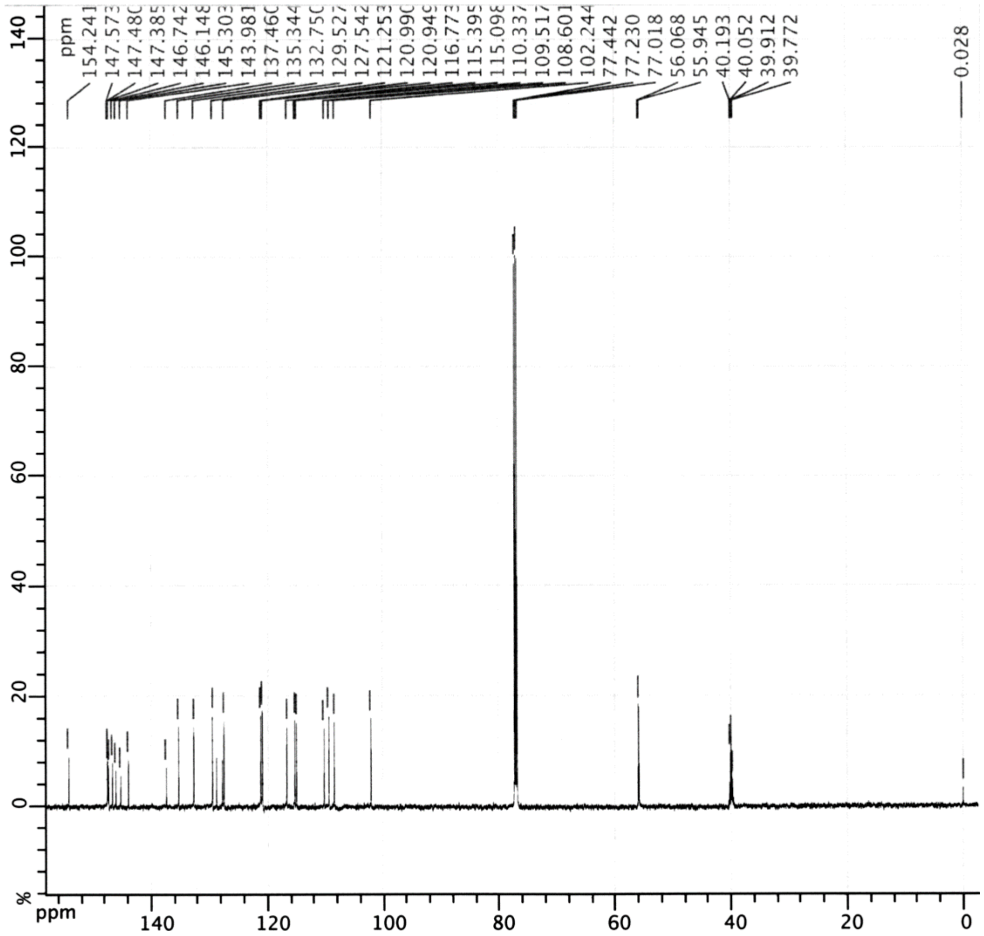

S26. NMR ^{13}C spectrum of compound **9** recorded in a Varian 600 MHz and $CDCl_3$ as solvent.

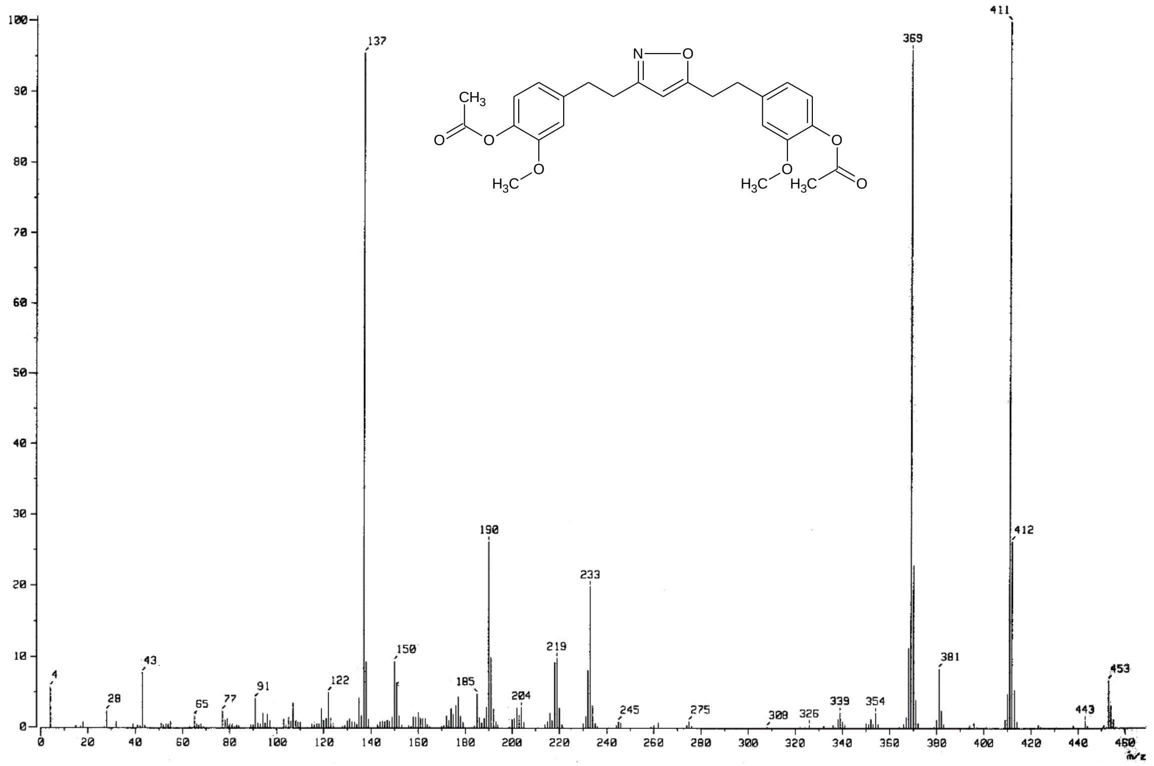

S27. Mass spectrum of compound **10** recorded in a Jeol JMS-AX505HA equipment by direct inlet and EI mode ion detection.

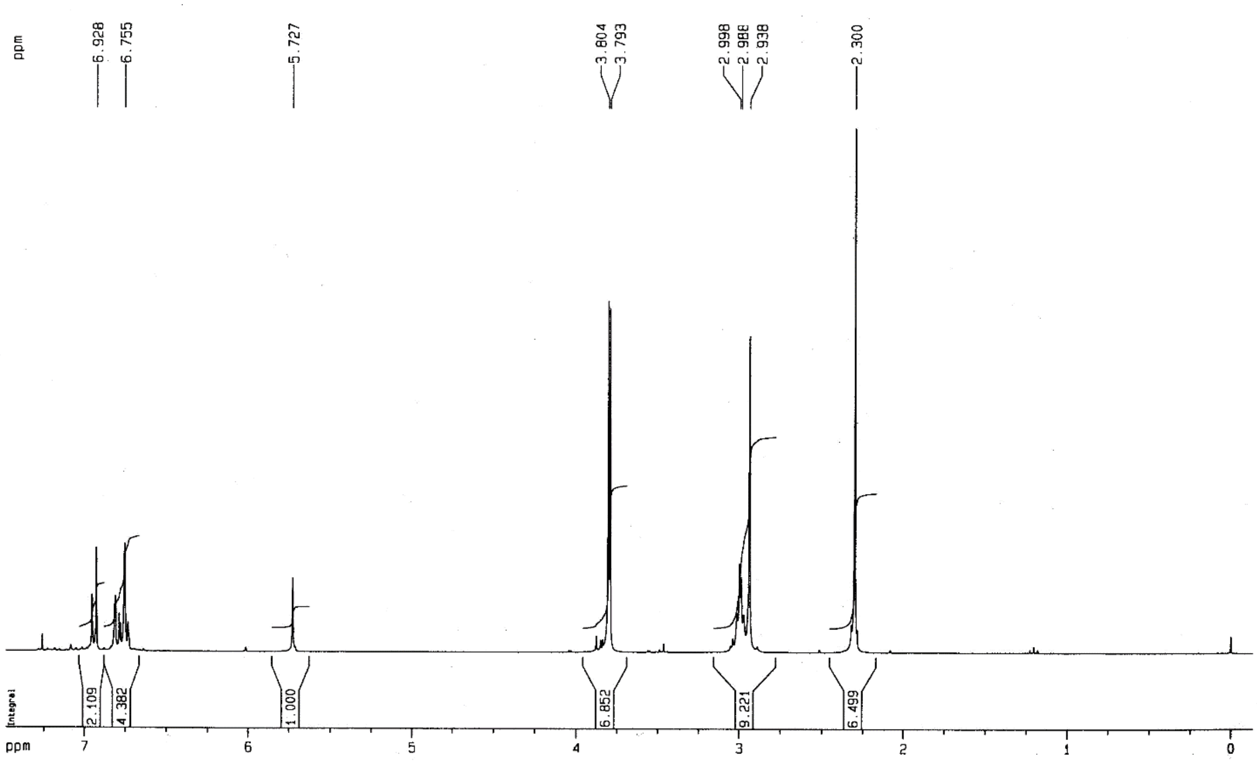

S28. NMR ^1H spectrum of compound **10** recorded in a Bruker 300 MHz and $CDCl_3$ as solvent.

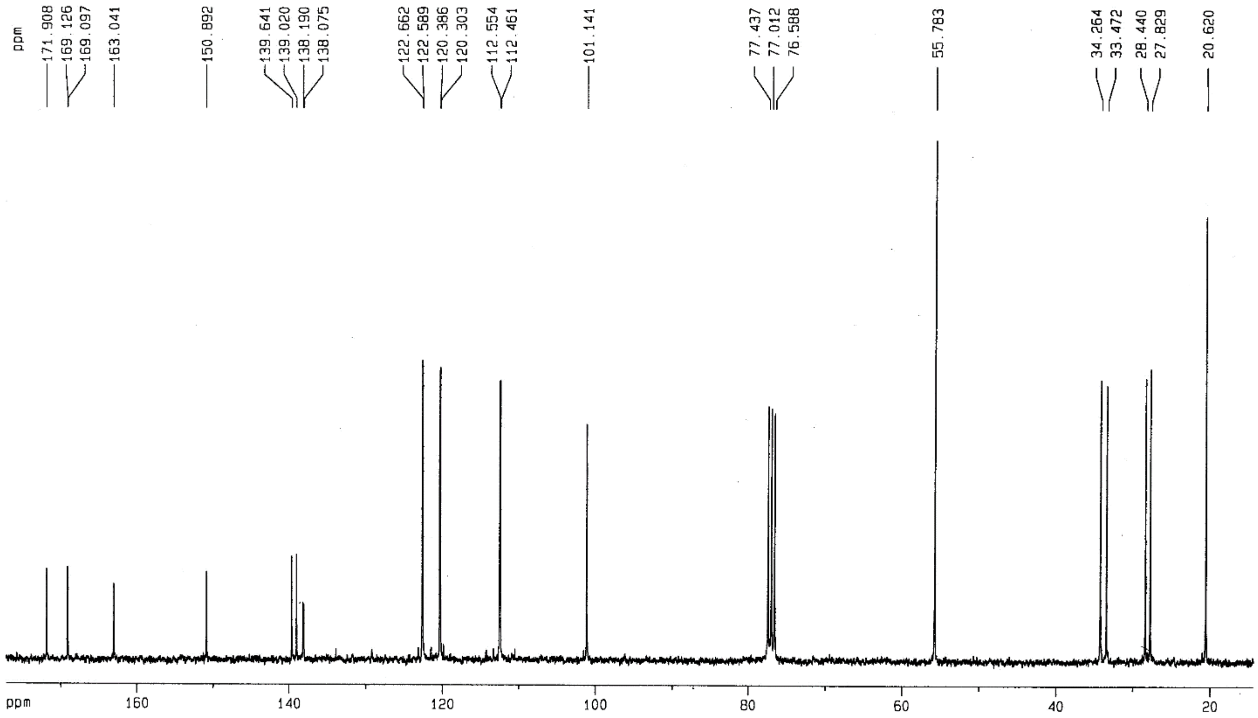

S29. NMR ^{13}C spectrum of compound **10** recorded in a Bruker 300 MHz and $CDCl_3$ as solvent.

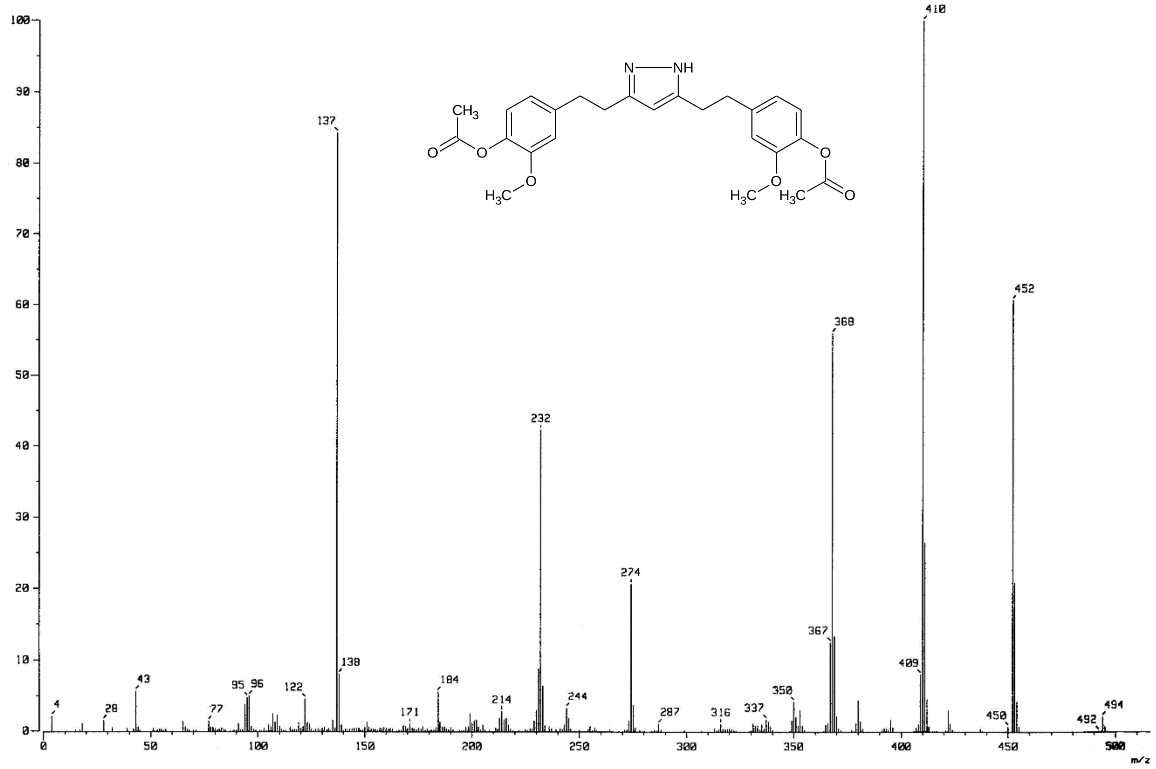

S30. Mass spectrum of compound **11** recorded in a Jeol JMS-AX505HA equipment by direct inlet and EI mode ion detection.

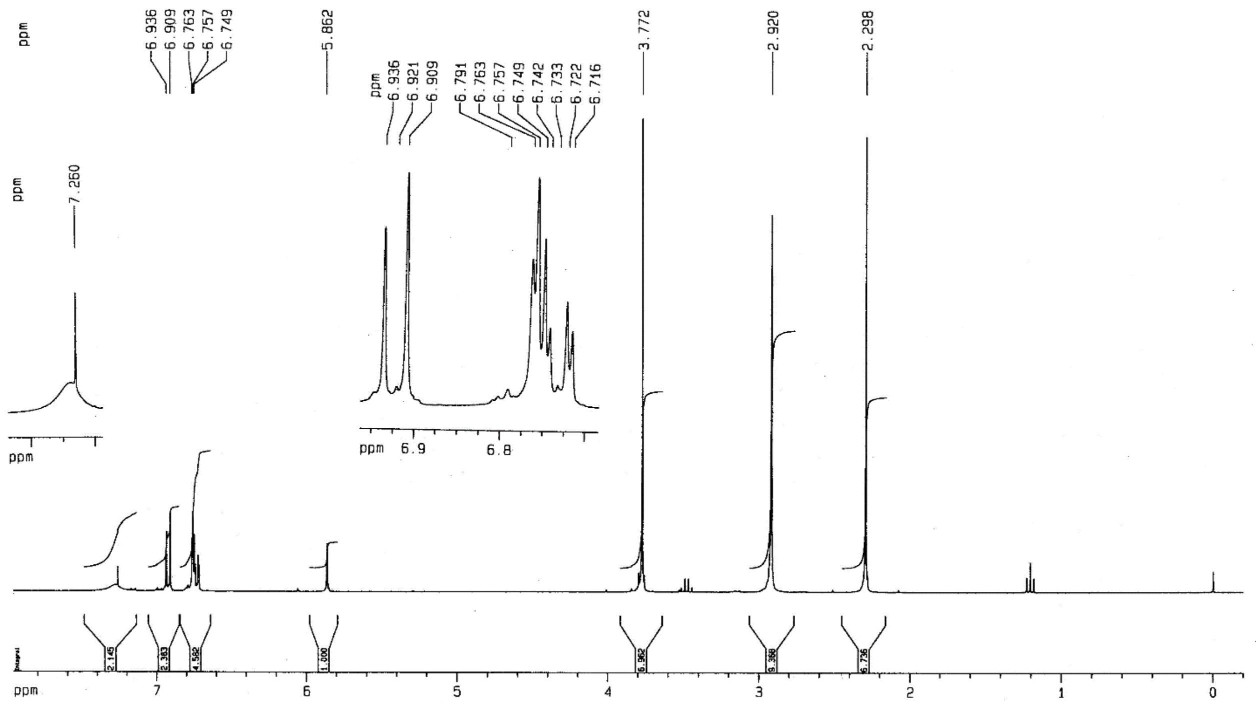

S31. NMR ^{1}H spectrum of compound **11** recorded in a Bruker 300 MHz and $CDCl_3$ as solvent.

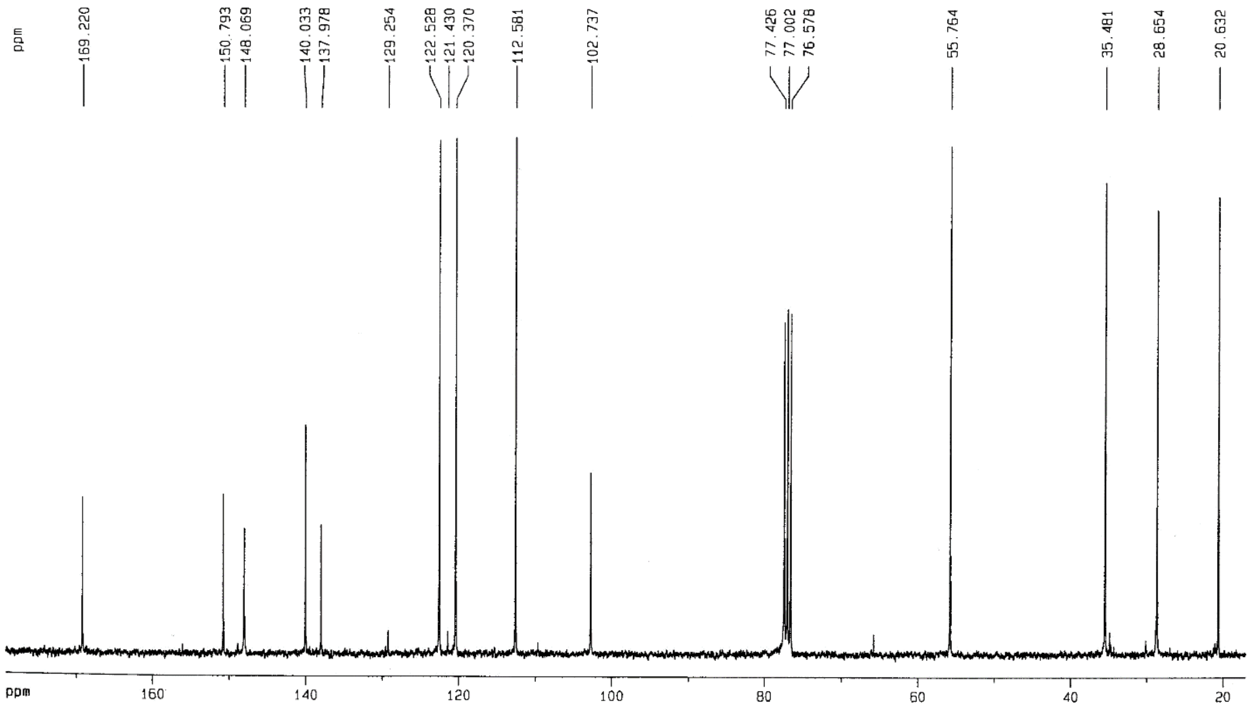

S32. NMR ^{13}C spectrum of compound **11** recorded in a Bruker 300 MHz and $CDCl_3$ as solvent.
